# MLatom 2: An Integrative Platform for Atomistic Machine Learning

**DOI:** 10.1007/s41061-021-00339-5

**Published:** 2021-06-08

**Authors:** Pavlo O. Dral, Fuchun Ge, Bao-Xin Xue, Yi-Fan Hou, Max Pinheiro, Jianxing Huang, Mario Barbatti

**Affiliations:** 1 State Key Laboratory of Physical Chemistry of Solid Surfaces, Fujian Provincial Key Laboratory of Theoretical and Computational Chemistry, Xiamen, 361005 China; 2 Department of Chemistry, and College of Chemistry and Chemical Engineering, Xiamen University, Xiamen, 361005 China; 3 Aix Marseille University, CNRS, ICR, Marseille, France

**Keywords:** Machine learning, Quantum chemistry, Kernel ridge regression, Neural networks, Gaussian process regression

## Abstract

Atomistic machine learning (AML) simulations are used in chemistry at an ever-increasing pace. A large number of AML models has been developed, but their implementations are scattered among different packages, each with its own conventions for input and output. Thus, here we give an overview of our MLatom 2 software package, which provides an integrative platform for a wide variety of AML simulations by implementing from scratch and interfacing existing software for a range of state-of-the-art models. These include kernel method-based model types such as KREG (native implementation), sGDML, and GAP-SOAP as well as neural-network-based model types such as ANI, DeepPot-SE, and PhysNet. The theoretical foundations behind these methods are overviewed too. The modular structure of MLatom allows for easy extension to more AML model types. MLatom 2 also has many other capabilities useful for AML simulations, such as the support of custom descriptors, farthest-point and structure-based sampling, hyperparameter optimization, model evaluation, and automatic learning curve generation. It can also be used for such multi-step tasks as Δ-learning, self-correction approaches, and absorption spectrum simulation within the machine-learning nuclear-ensemble approach. Several of these MLatom 2 capabilities are showcased in application examples.

## Introduction

Machine learning (ML) has taken computational chemistry by storm [1–4]. It is often applied to find a relationship between given molecular geometry and quantum chemical (QC) properties. A particularly useful application of such atomistic ML (AML) models is mapping molecular potential energy surfaces (PESs) [5–8]. Creating AML models is, however, a complicated task and requires domain knowledge. Thus, much effort has been put into developing a mathematical foundation and writing specialized software for such simulations.

One of us (POD) started to develop the MLatom program package [9, 10] for atomistic simulations with ML already in 2013 when not many such packages were available. At first, it was written entirely in Fortran and parallelized with OpenMP as a self-contained black-box program for user-friendly calculations. Now, this part comprises the Fortran core of MLatom called MLatomF. Later, MLatomF added a Python wrapper called MLatomPy implementing multi-step tasks such as Δ-learning [11] and self-correction [12]. We have implemented these and other methods developed by ourselves, such as structure-based sampling [12], the KREG model [12], ML-nuclear ensemble approach (ML-NEA) for precise absorption spectrum simulations [13], as well as selected literature methods, such as those based on the Coulomb matrix descriptor [14, 15], in MLatom for development purposes, tighter integration, and higher efficiency (see "Native Implementations"). We have used these native implementations also for developing methods for improving QC Hamiltonian [16], accelerating ML nonadiabatic excited-state dynamics [17], and for PES construction with spectroscopic accuracy by introducing a hierarchical ML (hML ) [18] approach.

In recent years, we have witnessed the rapid rise of many exciting new ML models [4, 5]. They are often designed for different applications ranging from very accurate ML PES trained on as few as a hundred molecular configurations of small- and medium-sized molecules [19, 20] to ML models trained on thousands or millions of points to be transferable to large molecules [21, 22]. Each has its own advantages and disadvantages. It is, therefore, highly desirable to be able to test different models before applying them to the problem at hand. This is, however, a formidable task because these models are scattered in many different software packages. Each has its own conventions for input and output.

We face the same problem in our research: when we want to test some promising ML model, there is often a high entry barrier for learning how to use the corresponding package. Sometimes the documentation is very poor, and only interaction with experienced users or developers enabled us to use some packages. Often, some critical functionality, such as hyperparameter optimization, is missing.

Thus, as a pragmatic solution, we have provided the community with an integrated platform based on MLatom that interfaces the selection of popular third-party software packages via MLatomPy written in Python 3.6 + [23, 24]. This platform is released as MLatom 2 with all Python interfaces available as open-source, free software for non-commercial use. Importantly, the same input and output structure can now be used for many state-of-the-art, third-party ML models (see Interfaces). We have implemented the interfaces with sGDML [19, 25] (symmetrized gradient-domain ML), GAP and QUIP (providing GAP [26]-SOAP [27] method), TorchANI [28] (providing ANI [21] methods), DeePMD-kit [29] (providing the DPMD [30] and DeepPot-SE [31] methods), and PhysNet [22] programs. This selection of methods covers popular representatives of various types of methods, ranging from those based on kernel methods (KMs) to neural networks (NNs). Our implementation also supports hyperparameter optimization using Bayesian methods with Tree-structured Parzen Estimator (TPE) [32] via the hyperopt [33] package.

The modular structure of MLatom allows easy extension to other models in the future, as it requires only writing a separate independent module for converting MLatom input to the input of the third-party software and parsing the latter’s output. A similar approach is also used for interfacing various QC software packages [34]. This differs, however, from an alternative approach where some packages offer only part of an ML model, e.g., only a descriptor of a molecule to be used as an input for ML, as in DScribe [35].

In the following, we provide an overview of MLatom 2 capabilities, and details of native implementations and interfaces. We also demonstrate the application of MLatom 2 to several typical AML simulation tasks (hyperparameter optimization and generation of learning curves), Δ-learning and structure-based sampling, and calculation of absorption spectra.

## Overview

**Fig. 1:**
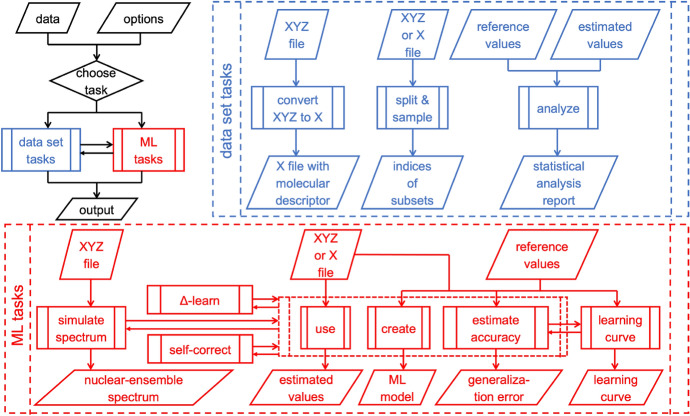
Overview of tasks performed by MLatom

The philosophy behind MLatom is to provide the community with a black-box-like program that allows a variety of calculation tasks required for ML atomistic simulations to be performed (Fig. 1). The program provides only with user-friendly and intuitive input, and no scripting is required. Under the hood, MLatom is built of modules designed to be independent of each other as much as possible to the extent that many modules can be used as stand-alone programs entirely independent from the main program. As needed, the modules are combined to create a seamless workflow eliminating step-by-step manual calculations.

The calculation tasks in MLatom are ML tasks and data set tasks. ML tasks are calculations involving training and using ML models. Our implementation includes a wide range of such tasks from basic to multi-step procedures: using an existing ML model from a file, creating an ML model and saving it to a file for later use, estimating ML model accuracy (model evaluation to determine the generalization error), Δ-learning [11], self-correction [12], learning curve and nuclear-ensemble spectrum generation [13]. Data set tasks perform all the operations necessary for ML simulations, such as converting geometries given in XYZ format to the input vector **x** for ML, splitting the data set into the required subsets (e.g., training, validation, and test), sampling points into these subsets, and performing statistical analysis of ML estimated values (e.g., error concerning reference values). These tasks can be performed either independently from each other, e.g., creating an ML model from available input vectors **x** or combined, e.g., by first converting XYZ coordinates to **x** and then creating an ML model. The user just needs to modify several input lines to perform simulations using the first or second option. In the following, we describe each of these tasks and define the most important concepts in ML atomistic simulations.

### ML Tasks

Currently, MLatom supports only supervised learning, which boils down to finding and using an approximating function $\widehat{f}\left(\mathbf{x};\mathbf{h};\mathbf{p}\right)$ that establishes a relationship between the reference values *y* and input vectors **x** in the training set based on statistically motivated assumptions [36] rather than on purely physical modelling. The approximating function typically has a large number of parameters **p** and so-called hyperparameters **h**.

#### Using ML Models

Using an existing ML model is conceptually simple as it requires information about the mathematical form of the approximating function and (hyper)parameters. It includes knowing how to transform a molecular geometry into an input vector **x**. One should pay attention, however, to many technical issues, such as ensuring consistent conventions for storing and retrieving this information from the file for long-term re-usability and reproducibility of scientific simulations. Another technical issue is related to performance and accuracy, as the information to be stored can be quite sizable, which can quickly lead to storage and input/output bottlenecks. MLatom saves ML model parameters and other information in a binary format file with a fixed structure for the core ML models and uses native formats of interfaced third-party software without converting them.

In atomistic simulations, we are also often interested in derivatives of properties. For example, in molecular dynamics simulations, we need to know derivatives of energy with respect to atomic coordinates (energy gradients =  − forces). Thus, MLatom can estimate both property values and partial derivatives with respect to elements of the input vector or atomic XYZ coordinates.

#### Creating ML Models

**Fig. 2:**
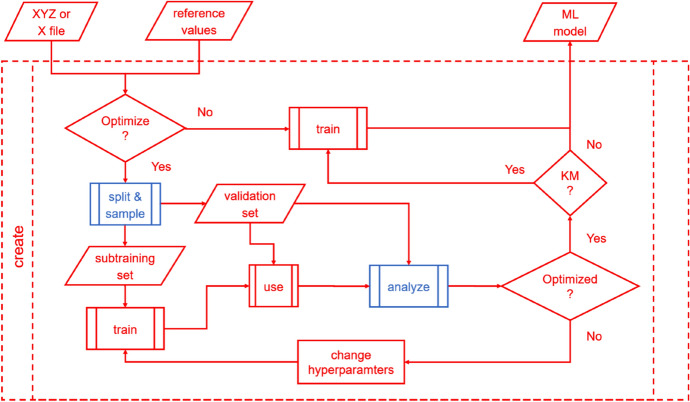
Creating a machine learning (ML) model with MLatom can involve automatic model selection (hyperparameter tuning) using different types of the training set split into sub-training and validation sets and different sampling procedures

Creating an ML model is already a much more complicated task as one needs to find the model (hyper)parameters in the right way (Fig. 2). This means that one needs to search for optimal values in the parameter space, leading to as low a generalization error as possible [36]. This is not the same as fitting parameters (training) that would give as low an error in the training set as possible. Modern ML methods can easily and exactly reproduce (an extreme case of overfitting) the reference values in the training set [2]. Thus, the standard practice is to set aside a validation set to ensure that training on the remaining data points will not lead to a large error in the validation set, i.e., to avoid overfitting [36]. The remaining data points are called either training or sub-training set in the literature, which adds to the confusion. While both conventions are valid, we prefer to call them sub-training points both here and in MLatom. All data points that are used in creating the ML model we call the training set. This set includes the sub-training and the validation sets in our nomenclature. This convention allows for a fairer and more straightforward comparison of ML models trained on the same number of training points as the validation set, which is used indirectly in training, to be accounted for. For example, if the model is trained on 1000 points, but used another 1000 points for validation, then the reference data is needed for all 2000 points, and such a model cannot be compared to another model trained on only 1000 points without using such a validation set.

When additional information such as derivatives of properties is available, it can be included into the training set too. It is common to train ML models for PESs simultaneously on energies and gradients (or only gradients), which is known to improve the quality of ML PESs significantly compared to fitting only on energies [7, 37, 38]. NNs simply fit parameters to the reference properties and their derivatives [38], while KMs can include the derivative information into their model explicitly [7, 37].

Many knobs exist and can be tuned in the process of finding suitable parameters for an ML model. One such knob concerns the ML model itself, and another deals with the splitting into sub-training and validation sets. As with the first type, while we do not need to touch the model parameters as this is the machine’s task, we can influence the model by changing its hyperparameters manually [36, 39]. As a side note, the difference between parameters and hyperparameters is somewhat fussy as the latter can be found by a machine too. Some hyperparameters also enter the ML model, while others do not. The external hyperparameters that do not enter the ML model are clearly different from parameters, but influence the training process, e.g., the regularization hyperparameter in KRR [36].

In any case, (hyper)parameters can be fitted to attempt to reduce the generalization error of the ML model by minimizing the error in the validation set (Fig. 2). For so-called parametric models such as NNs, whose approximating function does not explicitly depend on the training points, finding (hyper)parameters reducing the validation error is usually the end of the story. However, nonparametric models such as kernel ridge regression (KRR) and Gaussian process regression (GPR) depend explicitly on the training points. In their case, not using the validation set for training the final ML model would lose valuable additional information available to the model and reduce its accuracy. Thus, after hyperparameters minimizing error in the validation set for models trained on the sub-training set are found (a procedure also known as model selection), MLatom uses them to train the final model on the whole training set.

During hyperparameter optimization in MLatom, by default, the root-mean-squared error (RMSE) is minimized, but the minimization of another type of error can be requested for native implementations. Alternatively, other defaults can be used by interfaces if they have their own hyperparameter optimization capabilities. When the ML model is trained on several different properties, the error (loss, *L*) should reflect the error for each of these properties. For example, for models trained on both property values and their derivatives, e.g., energies and energy gradients, the error in MLatom can be calculated as the sum of error for values (*L*_val_) and weighted error for gradients in XYZ coordinates (*L*_grxyz_) as typically done in the literature [37]:

1 $$L={L}_{val}+{{w}_{grxyz}L}_{grxyz}$$

Although this approach gives the user additional flexibility, it has a drawback in that one has to choose an arbitrary parameter ${w}_{grxyz}$. To eliminate this parameter, we introduce in MLatom the geometric mean of errors of different properties, which is used by default:

2 $$L=\sqrt{{L}_{val}{L}_{grxyz}}$$

The final model's accuracy also depends on how the training set is split into sub-training and validation sets; this topic is overviewed below in "Splitting and Sampling".

#### Estimating Accuracy of ML Models

**Fig. 3:**
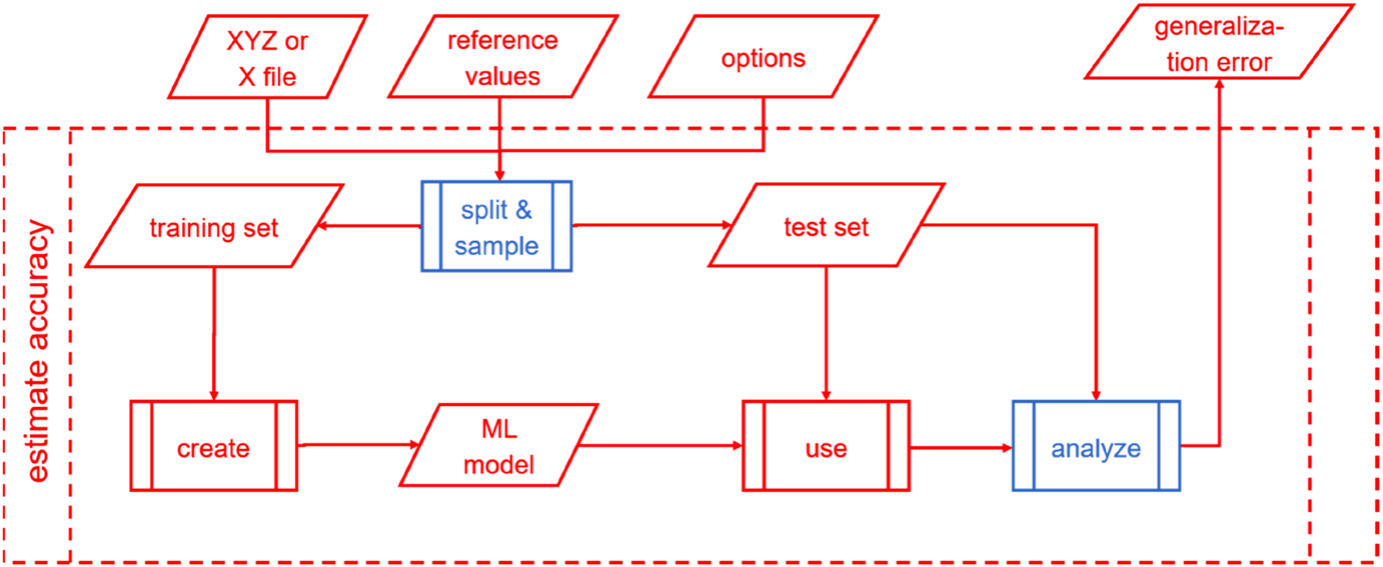
Estimating the accuracy of the ML model

MLatom also provides the means to estimate the accuracy (generalization error) of the ML model (Fig. 3). Since the validation set has already been used to create the ML model, it is good to use another independent test set to assess the model’s performance [36]. The entire data set can be split into training and test sets, and points can be sampled into these subsets using procedures similar to those used for hyperparameter tuning (model selection; see "Splitting and Sampling"). Naturally, hyperparameter tuning and model evaluation can be combined in a single calculation task with MLatom.

#### Multi-step Tasks

The tasks above can be considered as basic. We now turn to describe multi-step tasks built upon these basic tasks. One such task is a Δ-learning task that combines predictions at the low-level QC method with ML corrections to make estimations approaching high-level QC accuracy [11]. Another is a self-correction task that combines several layers of ML models, with each layer correcting the previous layer’s residual errors, which is useful for KRR models created with a focus on some region of the molecular PES [12]. Other multi-step tasks are learning curve generation and ML spectrum simulations [13], covered in the next two sub-sections in more detail.

#### Learning Curves

Here, the concept of learning curves is used to investigate how ML generalization error depends on training set size. The relationship between ML error ε and training set size typically follows the power-law decay [40]:

3 $$\varepsilon ={\varepsilon }_{\text{a}}+\frac{a}{{N}_{\text{tr}}^{b}}$$

where *ε*_a_ is the asymptotic error in the limit of the infinitely large training set, *a* is a nonnegative amplitude, and *b* is the positive exponent telling us how fast the ML improves by training with more data. These three parameters define the learning curve, giving a more complete characterization of the performance of a given ML model type than a single generalization error estimated for one training set size.

Since the errors drop that fast, the learning curves are often plotted on a log–log scale. In this case, particularly for not too large training sets and small asymptotic errors, a linear curve is often observed [37]:

4 $$\mathrm{log}\left(\varepsilon \right)\approx \mathrm{log}\left(a\right)-b\mathrm{log}{N}_{\text{tr}}$$

**Fig. 4:**
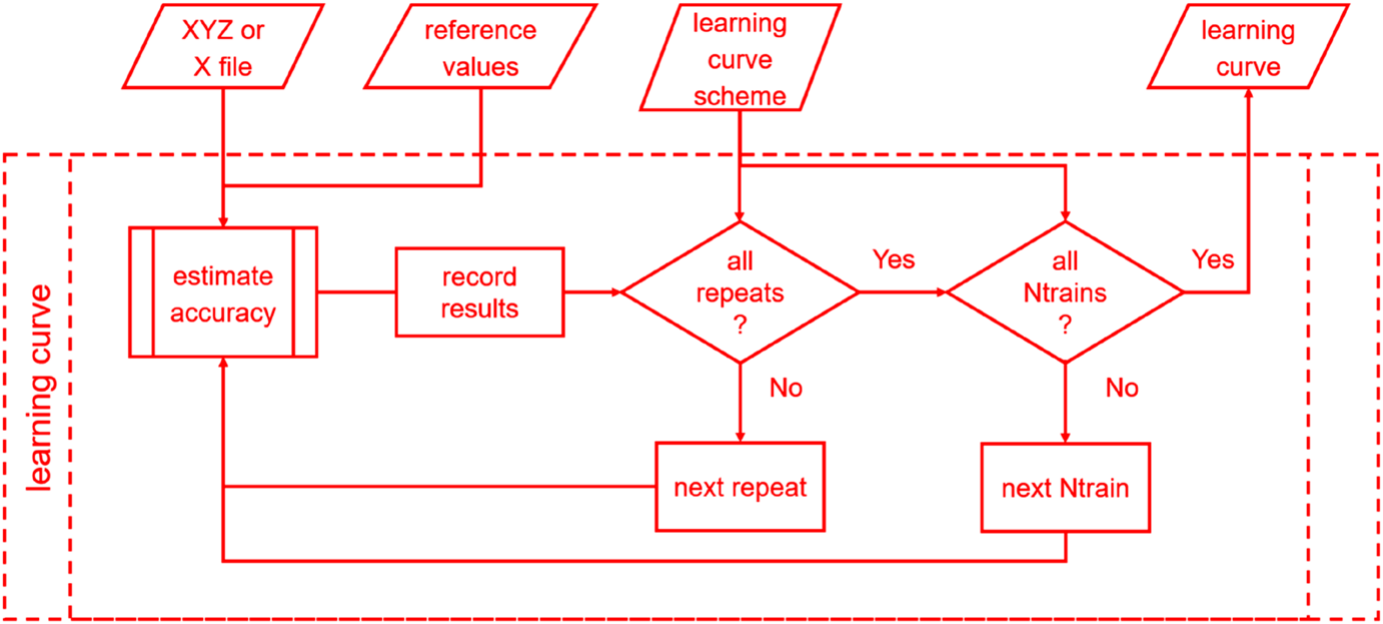
Flowchart for the learning curve task

A learning curve cannot be drawn without the accuracy-estimating step (see "Estimating Accuracy of ML Models") being taken multiple times. Thus, we provide a dedicated task to automate this procedure (Fig. 4) in MLatom 2.

In the learning curve task, the accuracy of each training set size requested is examined in multiple repeats, where different training points are sampled. Testing with repeats helps to reduce the bias introduced by a specific combination of training and test sets, investigates the statistical variance, and reflects the robustness of an ML model. The results (RMSEs, wall-clock training and prediction times) from each test are recorded in the .csv database file.

#### ML Nuclear Ensemble Spectra

**Fig. 5:**
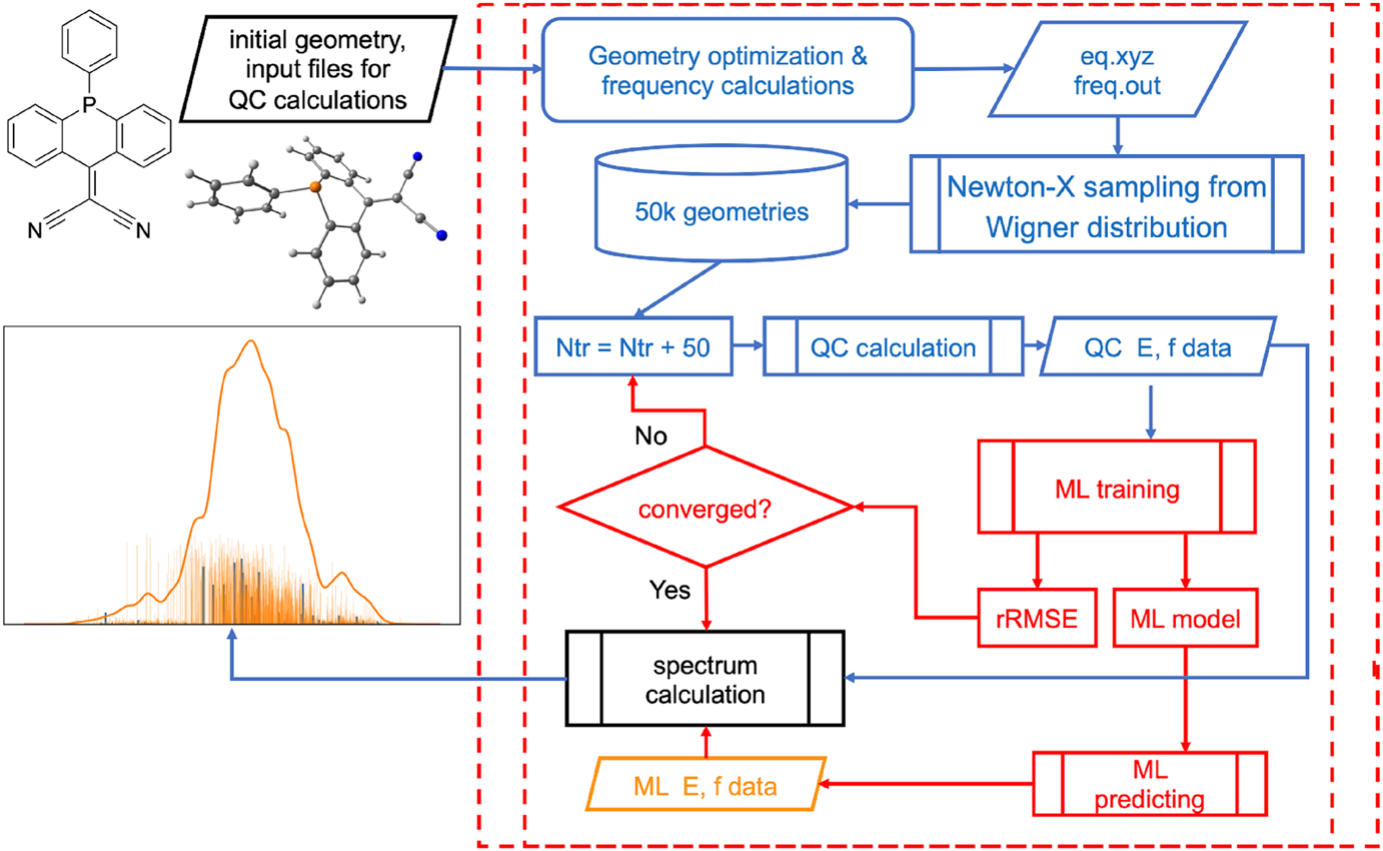
*Left* Schematic representation of the machine learning–nuclear ensemble approach (ML-NEA).* Right* Implementation of ML-NEA for calculating absorption spectra.* Blue* quantum chemical (QC) data,* orange* ML

Electronic spectrum simulation is yet another multi-step task that uses the ML-nuclear ensemble approach (ML-NEA) to automatically calculate quantities like absorption cross sections from as few QC calculations as possible [13]. This approach accelerates the traditional NEA, which usually requires thousands of points in a nuclear ensemble to generate a statistically converged spectrum [41]. Most nuclear ensemble points are relatively similar, making them suitable for using ML for efficient interpolation and replacing most QC calculations. Figure 5 shows this approach and its implementation in MLatom schematically.

The calculations require only an initial geometry, Gaussian 16 [42] input files for optimization and normal-mode calculations as well as for calculation of excited-state properties (excitation energies and oscillator strengths) with the QC method of choice. The user can also provide available pre-calculated data, such as output files with normal-mode calculations or nuclear ensemble geometries. Existing reference QC data can also be provided. MLatom has an interface to Gaussian 16, which automatically invokes and parses the QC calculations’ output to get the equilibrium geometry and frequencies. Then, it passes the required data to the interface to Newton-X [43, 44] to generate a nuclear ensemble of 50k conformations sampled from a harmonic-oscillator Wigner distribution of the nuclear coordinates [45].

QC properties for training ML models are calculated iteratively. The number of training points is increased at each iteration to train 2*N*_fs_ models for excitation energies (Δ*E*_0*n*_) and oscillator strengths (*f*_0*n*_) of transitions from ground-state to each excited state *n*. Then, we evaluate the convergence of ML predictions. We first calculate the geometric mean of the RMSE in the validation sets (${\mathrm{RMSE}}_{\mathrm{geom}}$) for all ML models, then take the relative change to the previous step (which had ${N}_{batch}=50$ fewer training points) to get the relative RMSE (rRMSE) as the convergence criterion (we consider it converged when rRMSE < 0.1):

5 $${\mathrm{RMSE}}_{\mathrm{geom}}(N)=\sqrt[2{N}_{fs}]{{\prod }_{i=1}^{{N}_{fs}}{\mathrm{RMSE}}_{{\Delta E}_{i}}\left(N\right)\bullet {RMS\mathrm{E}}_{{f}_{i}}(N)}$$

6 $$\mathrm{rRMSE}=\frac{{\mathrm{RMSE}}_{\mathrm{geom}}\left({N}_{\mathrm{tr}}\right)-{\mathrm{RMSE}}_{\mathrm{geom}}({N}_{\mathrm{tr}}-{N}_{batch})}{{\mathrm{RMSE}}_{\mathrm{geom}}({N}_{\mathrm{tr}})}$$

If the criterion is satisfied, we use current trained ML models to make predictions for the remaining nuclear ensemble points, substitute any negative ML oscillator strengths with zeros, and then calculate the absorption spectrum with the following equation [41]:

7 $$\sigma \left(E\right)=\frac{\pi {e}^{2}\hslash }{2mc{\varepsilon }_{0}E}{\sum }_{n}^{{N}_{fs}}\frac{1}{{N}_{p}}{\sum }_{i}^{{N}_{p}}\Delta {E}_{0n}\left({\mathbf{x}}_{i}\right){f}_{0n}\left({\mathbf{x}}_{i}\right)\frac{1}{\sqrt{2\pi {\left(\delta /2\right)}^{2}}}\mathrm{exp}\left(-\frac{{\left(E-\Delta {E}_{0n}\right)}^{2}}{2{\left(\delta /2\right)}^{2}}\right)$$

where *σ* is the absorption cross section, *m* and *e* are the electron mass and charge, $\hslash$ is the reduced Planck constant, *c* is the speed of light, ${\varepsilon }_{0}$ is the vacuum permittivity, ${N}_{p}$ is the number of ensemble points (50k by default), and $\delta =0.01$ eV is the broadening factor [13]. This summation can become quite computationally intensive for a large number of ensemble points; thus, we implemented it in C ++ . Currently, only absorption cross sections are available, but the code can be trivially adapted for other electronic spectrum types, like steady-state fluorescence.

### Data Set Tasks

The quality of ML models depends strongly on the descriptor, i.e., the chosen transformation of the molecular structure into the ML input vector **x**. While they are part of any ML model, MLatom currently only allows converting data sets with XYZ coordinates to the descriptors available in its native implementations (see "Native Implementations"). Other data set operations are discussed in the sub-sections below.

#### Splitting and Sampling

As we have seen, for tasks such as creating and estimating the accuracy of the ML model, the data set can be split into sub-sets: sub-training, validation, training, and test sets [36]. Data points can also be assigned to these sub-sets in different ways.

The simplest approach is to split the sets into sub-sets just once and sample the points randomly [10, 36]. When a data set to be explored with ML is known in advance, farthest-point (FPS) and structure-based sampling (SBS) [12] is possible and preferable to random sampling [10]. In both FPS and SBS, the data set points are sorted using an iterative procedure so that each next point is as far as possible from all the previous selected points [10]. The distance between points *i* and *j* is judged by the Euclidean distance ${\Vert {\mathbf{x}}_{i}-{\mathbf{x}}_{j}\Vert }_{2}$ between the corresponding descriptors, input vectors **x**_i_ and **x**_j_. In the case of SBS, the first point is the near-equlibrium geometry, in the case of FPS, the two most distant points are chosen as the first two points. Since these sampling procedures are based on an iterative greedy algorithm, they are implemented in MLatomF using Fortran and parallelized with OpenMP, which allows efficient sampling for data sets with tens of thousands of points [10]. SBS applied to sampling points from the PES of a single molecule would lead to underrepresentation of the near-equilibrium geometries as the most distorted geometries will be chosen. As a solution, the geometries are sorted by their Euclidean distance to the equilibrium geometry, sliced into regions corresponding to different degrees of deformation, and, finally, SBS can be performed from each of these regions to obtain a balanced set [2, 10, 12]. This slicing procedure is implemented in MLatomPy.

A more elaborate and slow technique for data set splitting is *k*-fold cross-validation, with leave-one-out cross-validation being the slowest [2, 10, 36]. In brief, the data set is split into *k* roughly equal parts, and then each of these parts is used for validation/testing, with the remaining parts used for training; after *k*-rotations of parts, the whole data set is effectively reused for validation/testing purposes. Thus, this procedure is useful for relatively small training sets.

All these techniques are available for native implementations of MLatom. User-defined sampling into these subsets can also be requested, and then the indices for each of the subsets should be provided to MLatom. By default, 80%:20% random splitting is used for native implementations, while interfaced third-party programs may use their default splitting and sampling for hyperparameter optimization and training (but not for model evaluation).

#### Analysis of Data

MLatom calculates several built-in statistical metrics for analyzing data, particularly for comparing ML estimations to the reference values. They are overviewed below for the sake of completeness. For *N* estimated values *ŷ* and reference values *y*:

- Mean absolute error (MAE):
  8 $${\text{MAE}}=\frac{1}{N}\sum_{i}^{N}\left|{\widehat{y}}_{i}-{y}_{i}\right|$$
- Mean signed error (MSE, not to be confused with mean squared error):
  9 $${\text{MSE}}=\frac{1}{N}\sum_{i}^{N}\left({\widehat{y}}_{i}-{y}_{i}\right)$$
- Root-mean-squared error (RMSE):
  10 $${\text{RMSE}}=\sqrt{\frac{1}{N}\sum_{i}^{N}{\left({\widehat{y}}_{i}-{y}_{i}\right)}^{2}}$$
- Arithmetic means of estimated and reference values, respectively:
  11 $${\mu }_{\widehat{y}}=\frac{1}{N}\sum_{i}^{N}{\widehat{y}}_{i}$$
  12 $${\mu }_{y}=\frac{1}{N}\sum_{i}^{N}{y}_{i}$$
- Largest positive and negative outliers as judged by $\widehat{y}-y$.
- Linear regression coefficients *a* and *b* in *ŷ* = *a* + *by*, their standard errors (SEs) with the corresponding correlation coefficient *R* and its squared value *R*^2^ found by least-squares fitting [46]:
  13 $$b=\frac{{\text{ss}}_{y\widehat{y}}}{{\text{ss}}_{yy}}$$
  14 $$a={\mu }_{\widehat{y}}-b{\mu }_{y}$$
  15 $${\text{SE}}\left(a\right)=s\sqrt{\frac{1}{N}+\frac{{\mu }_{y}^{2}}{{\text{ss}}_{yy}}}$$
  16 $${\text{SE}}\left(b\right)=\frac{s}{\sqrt{{\text{ss}}_{yy}}}$$
  17 $${R}^{2}=\frac{{ss}_{y\widehat{y}}^{2}}{{\text{ss}}_{yy}{\text{ss}}_{\widehat{y}\widehat{y}}}$$

where

18 $${\text{ss}}_{yy}=-N{\mu }_{y}^{2}+\sum_{i}^{N}{y}_{i}^{2}$$

19 $${\text{ss}}_{\widehat{y}\widehat{y}}=-N{\mu }_{\widehat{y}}^{2}+\sum_{i}^{N}{\widehat{y}}_{i}^{2}$$

20 $${\text{ss}}_{y\widehat{y}}=-N{\mu }_{y}{\mu }_{\widehat{y}}+\sum_{i}^{N}{{y}_{i}\widehat{y}}_{i}$$

21 $$s=\sqrt{\frac{{\text{ss}}_{\widehat{y}\widehat{y}}-{ss}_{y\widehat{y}}^{2}/{\text{ss}}_{yy}}{N-2}}$$

Analogous expressions are used for derived properties, such as partial derivatives; in the latter case, each partial derivative is treated as a data point, e.g. for RMSE of energy gradients $\frac{\partial E}{\partial M}$ in XYZ coordinates evaluated for the PES of a single molecule with *N*_at_ atoms:

22 $${\text{RMSE}}_{grxyz}=\sqrt{\frac{1}{N\bullet {N}_{at}\bullet 3}\sum_{i=1}^{N}\sum_{a=1}^{{N}_{\text{at}}}\sum_{t=1}^{3}{\left(\frac{\partial {\widehat{E}}_{i}}{\partial {M}_{i,at}}-\frac{\partial {E}_{i}}{\partial {M}_{i,at}}\right)}^{2}}$$

## Native Implementations

This section overviews the theory and provides technical details behind the native implementations available in MLatom. All native ML models are currently based on KRR, so this approach is described first, and then we describe the details behind the KREG model and approaches based on the Coulomb matrix. The code for the KREG model, hyperparameter grid search, farthest-point, and structure-based sampling was optimized for efficient computing, while no such efforts were necessarily made for other implementations.

### Kernel Ridge Regression

The approximating function $\widehat{f}\left(\mathbf{x};\mathbf{h};\mathbf{p}\right)$ in KRR is the sum over all training points *N*_tr_:[36]

23 $$\widehat{f}\left(\mathbf{x};\mathbf{h};\mathbf{p}\right)=\sum_{j=1}^{{N}_{\text{tr}}}{\alpha }_{j}k\left(\mathbf{x},{\mathbf{x}}_{j};\mathbf{h}\right),$$

where *k* is the kernel function, **p** are model parameters that include the set of the regression coefficients *α* and **h** are parameters present in the kernel function.

The regression coefficients are found by solving the linear system of equations regularized by adding a small, nonnegative constant value *λ* to the diagonal elements [36]:

24 $$\left(\begin{array}{ccc}k\left({{\varvec{x}}}_{1},{{\varvec{x}}}_{1}\right)+\lambda & \cdots & k\left({{\varvec{x}}}_{1},{{\varvec{x}}}_{{N}_{\text{tr}}}\right)\\ \vdots & \ddots & \vdots \\ k\left({{{\varvec{x}}}_{{N}_{\text{tr}}},{\varvec{x}}}_{1}\right)& \cdots & k\left({{\varvec{x}}}_{{N}_{\text{tr}}},{{\varvec{x}}}_{{N}_{\text{tr}}}\right)+\lambda \end{array}\right)\left(\begin{array}{c}{\alpha }_{1}\\ \vdots \\ {\alpha }_{{N}_{\text{tr}}}\end{array}\right)=\left(\begin{array}{c}{y}_{1}\\ \vdots \\ {y}_{{N}_{\text{tr}}}\end{array}\right)$$

or in matrix form:

25 $$\left(\mathbf{K}+\lambda \mathbf{I}\right){\varvec{\upalpha}}=\mathbf{y}$$

where **I** is the identity matrix, **K** is the kernel matrix that evaluates the kernel function for each pair of the training points, and **y** is the vector with reference values. λ is called the regularization parameter and is an external hyperparameter not entering the approximating function itself but used for model selection.

This system of equations has an analytical solution that makes it very attractive. The solution is, however, computationally costly for large data sets as it involves some kind of matrix decomposition that scales as *O*(*N*_tr_^3^), followed by solving the system of equations, which scales as *O*(*N*_tr_^2^). MLatom uses the very computationally efficient Cholesky decomposition by default. Bunch–Kaufman and LU decomposition approaches are also available, which are sometimes necessary when Cholesky decomposition fails [47]. The solution of the above system of equations also requires calculation of the kernel matrix of size *N*_tr_^2^, which can become very large and no longer fit in the available computer memory. Note that, by default, MLatom does not invert the matrix to solve the system of equations using the common expression [36]:

26 $${\varvec{\upalpha}}={\left(\mathbf{K}+\lambda \mathbf{I}\right)}^{-1}\mathbf{y}$$

as it is much less computationally efficient and numerically less precise [39].

The kernel functions supported by MLatom are [2, 10, 36, 39]:

- Gaussian:
  27 $$k\left(\mathbf{x},{\mathbf{x}}_{j}\right)=\mathrm{exp}\left(-\frac{1}{2{\sigma }^{2}}\sum_{s}^{{N}_{x}}{\left({x}_{s}-{x}_{j,s}\right)}^{2}\right)$$
- Laplacian:
  28 $$k\left(\mathbf{x},{\mathbf{x}}_{j}\right)=\mathrm{exp}\left(-\frac{1}{\sigma }\sum_{s}^{{N}_{x}}\left|{x}_{s}-{x}_{j,s}\right|\right)$$
- Exponential:
  29 $$k\left(\mathbf{x},{\mathbf{x}}_{j}\right)=\mathrm{exp}\left(-\frac{1}{\sigma }{\left[\sum_{s}^{{N}_{x}}{\left({x}_{s}-{x}_{j,s}\right)}^{2}\right]}^{1/2}\right)$$
- Matérn:
  30 $$k\left(\mathbf{x},{\mathbf{x}}_{j}\right)=\mathrm{exp}\left(-\frac{1}{\sigma }{\left[\sum_{s}^{{N}_{x}}{\left({x}_{s}-{x}_{j,s}\right)}^{2}\right]}^{1/2}\right)\sum_{k=0}^{n}\frac{\left(n+k\right)!}{\left(2n\right)!}\left(\begin{array}{c}n\\ k\end{array}\right){\left(\frac{2}{\sigma }{\left[\sum_{s}^{{N}_{x}}{\left({x}_{s}-{x}_{j,s}\right)}^{2}\right]}^{1/2}\right)}^{n-k}$$

where ${N}_{x}$ is the dimensionality of the input vector **x**, and the symbol for hyperparameters **h** entering the kernel function was dropped. All of them have the length-scale parameter σ, which is an internal hyperparameter. The Matérn kernel function has an additional integer hyperparameter *n*. The choice of the kernel function depends on the application, and it can be considered a hyperparameter itself, although MLatom does not automatically make this choice. MLatom performs automatic optimization of the hyperparameters λ and σ on the nested logarithmic grid [10]. Alternatively, the hyperparameters can be optimized using a third-party hyperopt package (see "Interfaces" and its subsection "Hyperopt" below).

As discussed above, we are often interested in the derivatives of properties. Once the KRR approximating function is trained on reference values of properties, it can be differentiated to obtain the required derivatives with respect to the *d* dimension of the input vector **x**:

31 $$\frac{\partial \widehat{f}\left(\mathbf{x}\right)}{\partial {x}_{d}}=\sum_{j=1}^{{N}_{\text{tr}}}{\alpha }_{j}\frac{\partial k\left(\mathbf{x},{\mathbf{x}}_{j}\right)}{\partial {x}_{d}}.$$

Thus, calculating the approximating function derivatives requires calculation of kernel function derivatives. The expressions for analytical first-order derivatives of the kernel functions are:

- Gaussian:
  32 $$\frac{\partial k\left({\mathbf{x},\mathbf{x}}_{j}\right)}{\partial {x}_{d}}=\frac{1}{{\sigma }^{2}}\left({x}_{j,d}-{x}_{d}\right)k\left({\mathbf{x},\mathbf{x}}_{j}\right)$$
- Matérn with *n* > 0:
  33 $$\frac{\partial k\left(\mathbf{x},{\mathbf{x}}_{j}\right)}{\partial {x}_{d}}=\mathrm{exp}\left(-\frac{{\Vert \mathbf{x}-{\mathbf{x}}_{j}\Vert }_{2}}{\sigma }\right)\sum_{k=0}^{n-1}\frac{\left(n+k-1\right)!}{\left(2n\right)!}\left(\begin{array}{c}n\\ k\end{array}\right)\times\left(n-k\right)\frac{2}{{\sigma }^{2}}{\left(\frac{2{\Vert \mathbf{x}-{\mathbf{x}}_{j}\Vert }_{2}}{\sigma }\right)}^{n-k-1}\left({x}_{j,d}-{x}_{d}\right)$$

where $\left\| {{\mathbf{x}} - {\mathbf{x}}_{j} } \right\|_{2} = \left[ {\sum\nolimits_{x}^{{N_{s} }} {(x_{s} - x_{{j,s}} )^{2} } } \right]^{{1/2}}$ is the Euclidean distance. The exponential and Laplacian kernel functions, as well as the Matérn kernel function with *n* = 0 are not differentiable.

Often, we need to know the derivatives in XYZ coordinates. Let us define the molecular XYZ coordinates as **M** that can be transformed into the input vector x via descriptor function *x*(**M**). Then, the partial derivatives in XYZ coordinates for atom *a* and dimension *t* can be obtained using the chain rule as:

34 $$\frac{\partial \widehat{f}\left(x\left(\mathbf{M}\right)\right)}{\partial {M}_{at}}=\sum_{j=1}^{{N}_{\text{tr}}}{\alpha }_{j}\sum_{d=1}^{{N}_{d}}\frac{\partial k\left(\mathbf{x},{\mathbf{x}}_{j}\right)}{\partial {x\left(\mathbf{M}\right)}_{d}}\frac{\partial {x\left(\mathbf{M}\right)}_{d}}{\partial {M}_{at}}$$

where *x*(**M**)_*d*_ are ${N}_{d}$ elements of the input vector **x** depending on *M*_at_. The expression for the first-order derivatives of the descriptors $\partial {x\left(\mathbf{M}\right)}_{d}/\partial {M}_{at}$ available in MLatom are given in the following sections.

Note that the derivative information can also be included in the training data for KRR, which usually requires implementing higher-order derivatives of the kernel functions [26, 37, 39, 47–50]. Such implementations in MLatom are currently underway and will be released in the near future.

All KRR calculations are implemented in Fortran and OpenMP, and we use the efficient Intel® Math Kernel Library (MKL) LAPACK[51] routines for calculating regression coefficients.

### KREG

KREG is the ML model type designed for constructing accurate PESs of a single molecule [12]. Its name is strictly speaking not an acronym but can be somewhat loosely derived from the first letters of the underlying components: KRR with the RE descriptor and the Gaussian kernel function. The RE descriptor is a vector of all inverse distances ${r}_{a,b\ne a}$ between atoms *a* and $b\ne a$ in a molecule normalized relative to the corresponding distances in some reference structure of the same molecule, usually its equilibrium geometry:

35 $${\mathbf{x}}^{\mathrm{T}}=\left[\begin{array}{ccc}\cdots & \frac{{r}_{a,b\ne a}^{\text{ref}}}{{r}_{a,b\ne a}}& \cdots \end{array}\right]$$

The RE descriptor is a global descriptor, meaning that it describes the molecule as a whole, and the KREG model learns the QC property directly without partitioning it into, e.g., atomic contributions as done by many models discussed below. This descriptor is also a complete descriptor, meaning that it uniquely represents the molecular geometry as the latter can always be derived from the descriptor up to rotation, translation, and such symmetry operations as reflection [10]. Rotational and translation invariance is a feature of the RE descriptor that ensures that scalar properties such as total energy are invariant to these operations according to the laws of physics [10].

The RE descriptor does not, however, ensure homonuclear permutational invariance, e.g., that interchange of hydrogen atoms in the methyl group CH_3_ does not change the total energy [10, 12]. Thus, several variants of the KREG model are possible depending on how this issue is dealt with. The simplest approximation is to neglect permutational invariance, i.e., by using the unsorted RE descriptor as obtained after transforming XYZ coordinates [10]. Another approximation is to sort homonuclear atoms using some criteria. In MLatom, this is done by sorting homonuclear atoms in descending order with respect to the sum of nuclear repulsions to other atoms [12]. This may help ensure the same sorting of atoms while doing structure-based sampling but may lead to discontinuities in the approximating function, which is problematic, e.g., in molecular dynamics [12]. Finally, permutational invariance can be ensured using the permutational invariant kernel that takes as input the permuted RE descriptor (see "Permutationally invariant kernel") [12]. The latter is the most accurate but also most computationally expensive [10].

If the derivatives of the KREG model are necessary, they can be easily obtained using the expressions discussed in the previous sub-section and the first-order derivative of the RE descriptor:

36 $$\frac{\partial {x\left(\mathbf{M}\right)}_{d}}{\partial {M}_{at}}={x}_{d}\frac{1}{{r}_{a,b}^{2}}\left({M}_{bt}-{M}_{at}\right),$$

where

37 $${x}_{d}=\frac{{r}_{a,b}^{\text{ref}}}{{r}_{a,b}}=\frac{{r}_{a,b}^{\text{ref}}}{\sqrt{\left(\sum_{s=1}^{3}{\left({M}_{as}-{M}_{bs}\right)}^{2}\right)}}$$

The first-order derivatives of the KREG models are implemented for both the unsorted and permuted RE descriptor variants.

### Coulomb Matrix

The Coulomb matrix (CM) descriptor is popular in ML studies, where its vectorized form is used as the input vector [14]:

$$\mathbf{x}=\mathbf{v}\mathbf{e}\mathbf{c}\left(\begin{array}{ccc}0.5{{\varvec{Z}}}_{1}^{2.4}& \cdots & \frac{{{\varvec{Z}}}_{1}{{\varvec{Z}}}_{{{\varvec{N}}}_{\text{at}}}}{{{\varvec{r}}}_{1{{\varvec{N}}}_{\text{at}}}}\\ \vdots & \ddots & \vdots \\ \frac{{{\varvec{Z}}}_{{{\varvec{N}}}_{\text{at}}}{{\varvec{Z}}}_{1}}{{{\varvec{r}}}_{{{\varvec{N}}}_{\text{at}}1}}& \cdots & 0.5{{\varvec{Z}}}_{{{\varvec{N}}}_{\text{at}}}^{2.4}\end{array}\right)$$

Like the RE descriptor, it is a global, complete descriptor based on the internuclear distances (in Bohr), ensuring rotational and translational invariance. However, it can also differentiate between molecules of different compositions by including nuclear charges *Z* (in a.u.) essentially to calculate internuclear repulsions in its off-diagonal elements. Its dimensionality, however, is limited by the largest number of ${N}_{\text{at}}$ atoms among molecules in the training set, and, for a smaller number of atoms, the CM descriptor elements are padded with zeros. Better ML models nowadays exist for treating molecules with a different number of atoms [4], and some of them are interfaced to MLatom, as discussed below. The CM matrix can also be used for constructing the PES of a single molecule though.

Like the RE descriptor, three variants of the CM matrix are available in MLatom: unsorted, sorted, and permuted. In the sorted variant, the atoms are sorted so that the Euclidean norms of columns and rows of the matrix are in descending order [15]. In the case of using the CM descriptor for a single molecule PES, the unsorted CM matrix has many redundant elements (as it is symmetric, only one-half of it is needed without diagonal elements), while the sorting can lead to large discontinuities in the approximating function [2]. Despite this, the unsorted Coulomb matrix was used in the molecular dynamics studies [52, 53], where its first-order derivative is also needed. It is implemented in MLatom for the unsorted variant as:

39 $$\frac{\partial {x\left(\mathbf{M}\right)}_{d}}{\partial {M}_{at}}=-\frac{{Z}_{a}{Z}_{b}}{{r}_{ab}^{3}}\left({M}_{at}-{M}_{bt}\right)$$

### Permutationally Invariant Kernel

Permutationally invariant (symmetrized) kernel is employed to take into account the permutational invariance of the homonuclear atoms [7, 10, 12, 18]:

40 $$\overline{k }\left(x\left(\mathbf{M}\right),x\left({\mathbf{M}}_{j}\right)\right)=\frac{{\sum }_{\widehat{P}}^{{N}_{perm}}k\left(x\left(\mathbf{M}\right),x\left(\widehat{P}{\mathbf{M}}_{j}\right)\right)}{\sqrt{{\sum }_{\widehat{P}}^{{N}_{perm}}k\left(x\left(\mathbf{M}\right),x\left(\widehat{P}\mathbf{M}\right)\right)}\sqrt{{\sum }_{\widehat{P}}^{{N}_{perm}}k\left(x\left({\mathbf{M}}_{j}\right),x\left(\widehat{P}{\mathbf{M}}_{j}\right)\right)}},$$

where *k* is one of the kernel functions mentioned above and $\widehat{P}$ permutes the order of atoms that are selected by the user. The descriptor is calculated for each such permutation, and hence it is called the permuted descriptor. The denominator normalizes the kernel function [39]. Unnormalized variants of this kernel symmetrization approach were used to extend the original GDML model type[48] to be permutationally invariant (creating the sGDML model type [19] interfaced to MLatom 2 and discussed below) and to create conceptually related RKHS + F [20] (reproducing kernel Hilbert space using energies and forces) model type.

The first-order derivative of the permutationally invariant kernel defined in Eq. 40 is given by:

41 $$\frac{\partial \overline{k }\left(x\left(\mathbf{M}\right),x\left({\mathbf{M}}_{j}\right)\right)}{\partial {M}_{at}}={\left[{\sum }_{\widehat{P}}^{{N}_{\mathrm{perm}}}k\left(x\left(\mathbf{M}\right),x\left(\widehat{P}\mathbf{M}\right)\right){\sum }_{\widehat{P}}^{{N}_{\mathrm{perm}}}k\left(x\left({\mathbf{M}}_{j}\right),x\left(\widehat{P}{\mathbf{M}}_{j}\right)\right)\right]}^{-1/2}\left\{\left[{\sum }_{\widehat{P}}^{{N}_{\mathrm{perm}}}\frac{\partial k\left(x\left(\mathbf{M}\right),x\left(\widehat{P}{\mathbf{M}}_{j}\right)\right)}{\partial {M}_{at}}\right]-\frac{1}{2}{\sum }_{\widehat{P}}^{{N}_{\mathrm{perm}}}\frac{\partial k\left(x\left(\mathbf{M}\right),x\left(\widehat{P}\mathbf{M}\right)\right)}{\partial {M}_{at}}\frac{{\sum }_{\widehat{P}}^{{N}_{\mathrm{perm}}}k\left(x\left(\mathbf{M}\right),x\left(\widehat{P}{\mathbf{M}}_{j}\right)\right)}{{\sum }_{\widehat{P}}^{{N}_{\mathrm{perm}}}k\left(x\left(\mathbf{M}\right),x\left(\widehat{P}{\mathbf{M}}_{i}\right)\right)}\right\}$$

The derivative $\frac{\partial k\left(x\left(\mathbf{M}\right),x\left(\widehat{P}{\mathbf{M}}_{j}\right)\right)}{\partial {M}_{at}}$ is analogous to the derivatives $\frac{\partial k\left(x\left(\mathbf{M}\right),x\left({\mathbf{M}}_{j}\right)\right)}{\partial {M}_{at}}$ shown above with the difference that elements ${x}_{j,d}$ change with each permutation as ${x\left(\widehat{P}{\mathbf{M}}_{j}\right)}_{d}$. The derivatives $\frac{\partial k\left(x\left(\mathbf{M}\right),x\left(\widehat{P}\mathbf{M}\right)\right)}{\partial {M}_{at}}$ require additional derivation because ${M}_{at}$ enters both $x\left(\mathbf{M}\right)$ and $x\left(\widehat{P}\mathbf{M}\right)$. The *d*th element stemming from atoms *a* and *b* in the original atom order corresponds to ${x\left(\mathbf{M}\right)}_{d}$ from which ${x\left(\widehat{P}\mathbf{M}\right)}_{d}$ is subtracted in both the Gaussian and Matérn kernel functions. On the other hand, the element stemming from atoms *a* and *b* in the permuted atom order will be ${x\left(\widehat{P}\mathbf{M}\right)}_{\widehat{P}d}={x\left(\mathbf{M}\right)}_{d}$, from which ${x\left(\mathbf{M}\right)}_{\widehat{P}d}$ is subtracted in both the Gaussian and Matérn kernel functions. Thus, the expressions for this term are for these kernel functions:

- Gaussian:
  42 $$\frac{\partial k\left(x\left(\mathbf{M}\right),x\left(\widehat{P}\mathbf{M}\right)\right)}{\partial {M}_{at}}=\frac{1}{{\sigma }^{2}}\left[\left({x\left(\mathbf{M}\right)}_{d}-{x\left(\widehat{P}\mathbf{M}\right)}_{d}\right)+\left({x\left(\mathbf{M}\right)}_{d}-{x\left(\mathbf{M}\right)}_{\widehat{P}d}\right)\right]k\left(x\left(\mathbf{M}\right),x\left(\widehat{P}\mathbf{M}\right)\right)$$
- Matérn:
  43 $$\frac{\partial k\left(x\left(\mathbf{M}\right),x\left(\widehat{P}\mathbf{M}\right)\right)}{\partial {M}_{at}}=\frac{2}{{\sigma }^{2}}\left[\left({x\left(\mathbf{M}\right)}_{d}-{x\left(\widehat{P}\mathbf{M}\right)}_{d}\right)+\left({x\left(\mathbf{M}\right)}_{d}-{x\left(\mathbf{M}\right)}_{\widehat{P}d}\right)\right]\times\mathrm{exp}\left(-\frac{{\Vert x\left(\mathbf{M}\right)-x\left(\widehat{P}\mathbf{M}\right)\Vert }_{2}}{\sigma }\right)\sum_{k=0}^{n-1}\frac{\left(n+k-1\right)!}{\left(2n\right)!}\times\left(\begin{array}{c}n\\ k\end{array}\right)\left(n-k\right){\left(\frac{2{\Vert x\left(\mathbf{M}\right)-x\left(\widehat{P}\mathbf{M}\right)\Vert }_{2}}{\sigma }\right)}^{n-k-1}$$

## Interfaces

As mentioned in the "Introduction", it is not that easy to start using a new ML model, especially for novices who did not get their feet wet in this field, even sometimes for experienced researchers who have some preconceptions from their familiar frameworks. Such difficulties could be alleviated by just implementing all models we want into a single all-in-one software. However, this approach is labor-intensive and unsustainable, considering the fast-growing numbers of ML models. A better solution to tackle this problem is to make interfaces to third-party software, which is easier to implement and modularize. The drawbacks of such interface-based solutions compared to all-in-one software are the need to install multiple third-party software packages and the decreased computational efficiency due to converting data and communication bottlenecks between programs. The interface-based approach has, however, the considerable benefit of the rapid development of one uniform workflow, which eliminates the problem at its origin: the lack of standardization. This allows researchers to quickly test multiple ML model types, an advantage that outweighs the drawbacks in many cases.

**Fig. 6:**
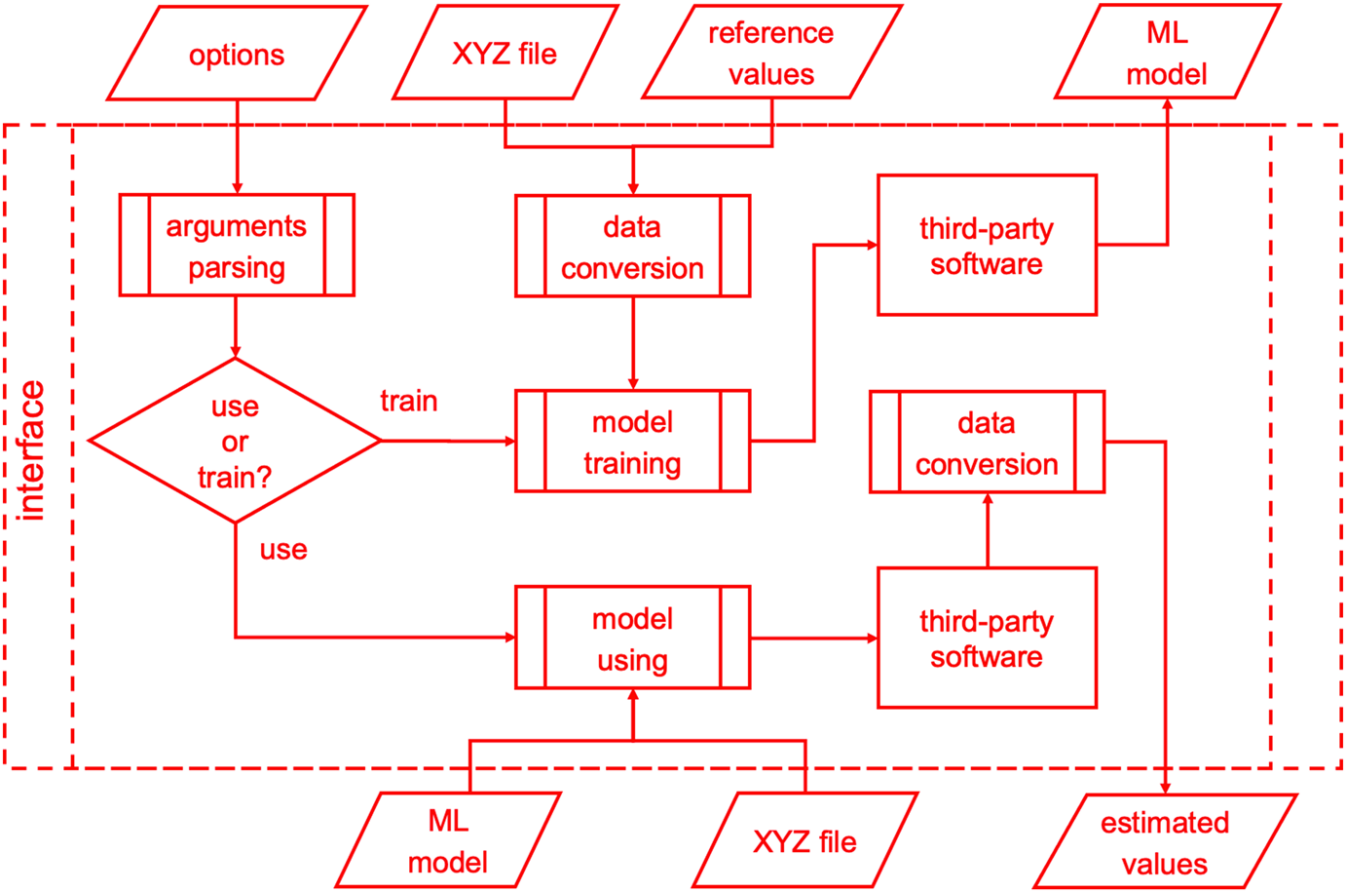
Flowchart for interfaces

Thus, we introduced the interfaces to third-party ML software in MLatom 2. Each interface should have four main functions inside, which are shown in Fig. 6. *Arguments parsing* translates MLatom-style input arguments to third-party equivalent. *Data conversion* takes in MLatom-format data then converts them into the corresponding format required by third-party software. *Model training* communicates with third-party software to get output models by sending prepared data and arguments. *Model using* uses third-party software to get estimated values from the trained model. The interfaces are modularized Python 3 scripts stored in the sub-folder *interfaces* in the MLatom program directory.

The currently interfaced third-party software packages are listed in Table 1 and will be introduced in the sub-sections below. Note that some programs provide several ML model types other than the listed ones. Using them through the interfaces might already be supported in MLatom or needs some slight modifications in the interface modules. However, only the listed model types are tested and will be discussed in this paper. Generally, we follow the strategy of making no modifications in the interfaced programs so that they can be taken “as is” and only the path to their executable should be known to MLatom. The only exception is the PhysNet, which uses TensorFlow v1. To make it compatible with the newer version of Python and TensorFlow [54], names of some legacy functions need to be changed to their equivalent in TensorFlow v2. Also, it is important to note that the main hyperparameters of each interfaced model can be modified easily through the standard input file of MLatom such that no modifications to the third-party software are required.

**Table 1:** Interfaced third-party software with their versions and ML model types tested here

Program (version)	ML model types	Developers	Language	URL
sGDML [25] (0.4.4)	sGDML [19]	Chmiela et al	Python	www.sgdml.org
GAP (1598976566) QUIP (5c61598e4)	GAP [26]-SOAP [27]	Csányi, Bartók, Kermode et al	Fortran	www.libatoms.org github.com/libAtoms/QUIP
TorchANI [28] (2.2)	ANI [21]	Gao, Ramezanghorbani, Smith, Isayev, Roitberg, et al	Python	github.com/aiqm/torchani
DeePMD-kit [29] (v1.2.2)	DPMD [30] DeepPot-SE [31]	Wang, Zhang, Han, E et al	C + + Python	www.deepmd.org/ github.com/deepmodeling/deepmd-kit
PhysNet [22]	PhysNet [22]	Unke, Meuwly	Python	github.com/MMunibas/PhysNet

Main program developers, programming languages of the majority of the code, and URL addresses to access the program are provided. References to ML model types (and third-party packages themselves where available) are also given

Besides the third-party ML software, we also implemented the interface to a hyperparameter optimization library called hyperopt [33], to provide a solution for hyperparameter optimization, which is unavailable in most of third-party software packages.

### Hyperopt

Hyperparameters—the parameters that tune the shape of the ML model and stay unchanged in the training—have a huge impact on the performance of an ML model. To unleash the full potential of an ML model, the hyperparameters need to be well optimized, and here comes the hyperparameter optimization problem. Solving this problem manually is too cumbersome and would rarely lead to the optimal solution. Hence, many packages that are capable of automatic hyperparameter optimization are available; hyperopt [33] is one of them. Hyperopt is an open-source Python library that uses a Bayesian method with tree-structured Parzen estimator (TPE) [32] to find the optima in hyperparameter spaces.

In MLatom 2, we added the interface that uses the hyperopt library as a convenient solution to the hyperparameter optimization problem. By simply substituting hyperparameters that need to be tuned with keywords for hyperopt search space, the interface will be activated to perform the automatic optimizing process (see Fig. 2). Optimization splits the training set into the sub-training and validation sets. The trained model’s error on the validation set will be sent to hyperopt to get the next searching point in hyperparameter space. If KMs are used, the final model will be generated by training on the entire training set with optimized hyperparameters after the optimization, while for NNs, the best model trained during the hyperparameter optimization will be the final ML model.

### sGDML

sGDML [19] is a symmetrized variant of the gradient-domain machine learning (GDML [48]) model type and is interfaced to MLatom through the sGDML program [25]. The sGDML method is a KRR model with the descriptor of unnormalized inverse distances (the ID descriptor)

44 $${\mathbf{x}}^{\mathrm{T}}=\left[\begin{array}{ccc}\cdots & \frac{1}{{r}_{a,b}}& \cdots \end{array}\right]$$

Thus, it has the same basic properties as the RE descriptor, i.e., the ID descriptor is also a global and complete descriptor ensuring rotational and translational invariance, but not permutational invariance of homonuclear atoms.

 In contrast to the KREG model, the GDML model learns only from the energy gradients. It also uses the Matérn kernel function, whose expression differs slightly from that implemented in MLatom. For learning from gradients, the common KRR approximating function for scalar properties is modified by using covariance between derivatives to predict the XYZ components of energy gradients:

45 $$\frac{\partial f\left(\mathbf{x}\right)}{\partial {M}_{\mathrm{at}}}=\sum_{j=1}^{{N}_{\text{tr}}}\sum_{b=1}^{{N}_{\text{at}}}\sum_{u=1}^{3}{\alpha }_{j,bu}\frac{{\partial }^{2}k\left(\mathbf{x},{\mathbf{x}}_{j}\right)}{\partial {M}_{\mathrm{at}}\partial {M}_{j,bu}}.$$

As usual, the linear system of KRR equations is solved with the kernel matrix **K**, now of a size $3{N}_{\text{at}}{N}_{\text{tr}}\times 3{N}_{\text{at}}{N}_{\text{tr}}$:

46 $$\left(\begin{array}{ccc}\frac{{\partial }^{2}k\left({\mathbf{x}}_{1},{\mathbf{x}}_{1}\right)}{\partial {M}_{\mathrm{1,11}}\partial {M}_{\mathrm{1,11}}}+\lambda & \cdots & \frac{{\partial }^{2}k\left({\mathbf{x}}_{1},{\mathbf{x}}_{{N}_{\text{tr}}}\right)}{\partial {M}_{\mathrm{1,11}}\partial {M}_{{N}_{\text{tr}},{N}_{\text{at}}3}}\\ \vdots & \ddots & \vdots \\ \frac{{\partial }^{2}k\left({\mathbf{x}}_{{N}_{\text{tr}}},{\mathbf{x}}_{1}\right)}{\partial {M}_{{N}_{\text{tr}},{N}_{\text{at}}3}\partial {M}_{\mathrm{1,11}}}& \cdots & \frac{{\partial }^{2}k\left({\mathbf{x}}_{{N}_{\text{tr}}},{\mathbf{x}}_{{N}_{\text{tr}}}\right)}{\partial {M}_{{N}_{\text{tr}},{N}_{\text{at}}3}\partial {M}_{{N}_{\text{tr}},{N}_{\text{at}}3}}+\lambda \end{array}\right)\left(\begin{array}{c}{\alpha }_{\mathrm{1,11}}\\ \vdots \\ {\alpha }_{{N}_{\text{tr}},{N}_{\text{at}}3}\end{array}\right)=\left(\begin{array}{c}\frac{\partial {y}_{1}}{\partial {M}_{\mathrm{1,11}}}\\ \vdots \\ \frac{\partial {y}_{{N}_{\text{tr}}}}{\partial {M}_{{N}_{\text{tr}},{N}_{\text{at}}3}}\end{array}\right).$$

When the energies are needed, they are obtained by integrating the energy gradients, and the integration constant is found by fitting to the reference energy values:

47 $$f\left(\mathbf{x}\right)=const+\sum_{j=1}^{{N}_{\text{tr}}}\sum_{b=1}^{{N}_{\text{at}}}\sum_{u=1}^{3}{\alpha }_{j,bu}\frac{\partial k\left(\mathbf{x},{\mathbf{x}}_{j}\right)}{\partial {M}_{j,bu}}.$$

The sGDML tackles the permutational invariance by using a modified, unnormalized permutational invariant kernel with permutations chosen automatically to minimize the matching cost between pairs of training points. The sGDML program also supports automatic hyperparameter optimization via cross-validation.

The sGDML method achieves remarkable accuracy given only a very small number of training points, as was shown for molecular PESs visited during dynamics [19]. Because of the large size of the kernel matrix, the method is practically applicable only for hundreds, up to several thousand training points. Note that when we refer to a single “training point,” we mean all the associated information for one geometry, and the real number of reference values available for sGDML is ${3N}_{\text{at}}$ times larger (the number of Cartesian energy gradient components). sGDML efficiently utilizes all this available information, which explains its accuracy.

The sGDML program requires a proprietary data format that uses NumPy’s [55] npz file as the container. Scripts to convert from other data formats (e.g., extended XYZ) are included in the program. Like MLatom, sGDML has a built-in hyperparameter optimization function for the hyperparameter $\sigma$ using a grid search, which is enabled by default; $\lambda$ is not optimized. The users can also specify $\sigma$ or a list of $\sigma$ values for the grid search, but only integers are acceptable.

### GAP-QUIP

The Gaussian approximation potential [26] (GAP) model is interfaced to MLatom through QUIP and GAP suite programs. Like native implementations in MLatom, GAP is also based on a kernel method, although it was developed within Gaussian process regression (GPR) formalism rather than KRR. In the GAP model, the total energy of a system is represented as the sum of atomic energies:

48 $$E=\sum_{i}^{\mathrm{atoms}}{\varepsilon }_{i}$$

As a result, local descriptors, rather than global descriptors as used in MLatom’s native models and sGDML, describing atomic environments for every single atom are used. The GAP suite provides a smooth overlap of atomic positions [27] (SOAP) descriptor for this purpose. The construction of a SOAP descriptor is quite involved as it has to respect all the required symmetries (rotational, translational, permutational), and its derivations are given in the literature [7, 27, 56]. Alternative versions of SOAP descriptor also exist [35].

Here, we describe the main points behind this descriptor. The local environment of an atom is represented by the atomic neighborhood density ${\rho }_{i}\left(\mathbf{r}\right)$ that is constructed using Gaussians for the *i*th atom as:

49 $${\rho }_{i}\left(\mathbf{r}\right)=\sum_{j}\mathrm{exp}\left(-\frac{{\left|\mathbf{r}-{\mathbf{r}}_{ij}\right|}^{2}}{2{\sigma }_{atom}^{2}}\right){f}_{\text{cut}}\left(\left|{\mathbf{r}}_{ij}\right|\right),$$

where ${\mathbf{r}}_{ij}$ is a vector pointing from atom *i* to the neighboring atom *j*, ${\upsigma }_{\text{atom}}$ reflects the atom “size”, and ${f}_{\text{cut}}$ is the cutoff function with the width of the cutoff region ${r}_{\Delta }$ approaching the limits of ${r}_{\text{cut}}$

50 $${f}_{\text{cut}}\left(r\right)=\left\{\begin{array}{cc}1,& r\le {r}_{\text{cut}}-{r}_{\Delta },\\ \frac{1}{2}\left(\mathrm{cos}\left(\pi \frac{r-{r}_{\text{cut}}+{r}_{\Delta }}{{r}_{\Delta }}\right)+1\right),& {r}_{\text{cut}}-{r}_{\Delta }<r\le {r}_{\text{cut}},\\ 0,& r>{r}_{\text{cut}},\end{array}\right.$$

The Gaussians are expanded with a set of orthonormal radial basis functions ${g}_{n}$ [57]:

51 $${\rho }_{i}\left(\mathbf{r}\right)=\sum_{\begin{array}{c}n<{n}_{\text{max}}\\ l<{l}_{\text{max}}\\ \left|m\right|\le l\end{array}}{c}_{nlm}^{i}{g}_{n}\left(r\right){Y}_{lm}\left(\widehat{\mathbf{r}}\right).$$

where $\widehat{\mathbf{r}}$ projects the direction of the vector** r** on the unit sphere and ${Y}_{lm}$ are spherical harmonics. For better efficiency, the choices of $n$ and $l$ are limited by ${n}_{\text{max}}$ and ${l}_{\text{max}}$, respectively. The orthonormal radial basis functions are constructed from

52 $${\phi }_{n}\left(r\right)=exp\left(-\frac{{\left(r-\frac{{r}_{\text{cut}}n}{{n}_{\text{max}}}\right)}^{2}}{2{\sigma }_{atom}^{2}}\right)$$

and the overlap matrix $\mathbf{S}={\mathbf{U}}^{T}\mathbf{U}$ with elements $S_{{nn^{\prime}}} = \int_{0}^{{r_{{{\text{cut}}}} }} {\phi_{n} (r)} \phi_{{n^{\prime}}} (r)r^{2} dr$ as

53 $$g_{n} (r) = \sum\nolimits_{{n^{\prime}}} {({\mathbf{U}}^{ - 1} )}_{{nn^{\prime}}} \phi_{{n^{\prime}}} (r).$$

The coefficients ${c}_{nlm}$ are obtained as

54 $${c}_{nlm}^{i}=\langle {g}_{n}\left.{Y}_{lm}\right|{\rho }_{i}\rangle .$$

The SOAP descriptor **p**_i_ consists of $\sum\nolimits_{m} {\left( {c_{{nlm}}^{i} } \right)} ^{ * } c_{{n^{\prime}lm}}^{i}$, which correspond to the power spectrum elements.

The kernel matrix elements are calculated using the dot-product kernel function

55 $${K}_{ij}=\left|{\mathbf{p}}_{i}\bullet {\mathbf{p}}_{j}\right|,$$

which are subsequently normalized, raised to a power of $\zeta$ (a positive integer) to tune its sensitivity, and scaled by ${\upsigma }_{\omega }$:

56 $${\overline{K} }_{ij}={\upsigma }_{\omega }{\left(\frac{{K}_{ij}}{\sqrt{{K}_{ii}}\sqrt{{K}_{jj}}}\right)}^{\zeta }.$$

Then the SOAP descriptor and kernel are used in estimating what the values of atomic energies are most likely to be by performing GPR that uses the same expression for making estimations as KRR:

57 $$\varepsilon \left(\mathbf{p}\right)=\sum_{j}{\alpha }_{j}K\left(\mathbf{p},{\mathbf{p}}_{j}\right)$$

The problem with this expression is that the *α* coefficients cannot be obtained directly using the similar expression as described in "Kernel ridge regression", because there are (usually) no reference *N*_tr_ atomic energies ${\varvec{\upvarepsilon}}$ and only *N*_obs_ total energies **E** are available. In the GAP approach, this is solved by introducing a linear transformation using the *N*_tr_ × *N*_obs_ matrix **L** with elements 1 or 0 so that

58 $$\mathbf{E}={\mathbf{L}}^{T}{\varvec{\upvarepsilon}},$$

Then, the kernel matrix becomes

59 $${\mathbf{K}}_{{N}_{\mathrm{obs}}{N}_{\mathrm{obs}}}={\mathbf{L}}^{T}{\mathbf{K}}_{{N}_{\mathrm{tr}}{N}_{\mathrm{tr}}}\mathbf{L},$$

Using this kernel matrix, the regression coefficients α can be calculated in the usual manner as in the KRR approach. In the GAP–SOAP notation, the regularization hyperparameter is denoted ${\sigma }_{E}^{2}$ for energies. The GAP–QUIP implementation also allows for using sparsification to reduce the size of the kernel matrix and, in this case, additional parameters defining the size ${N}_{\text{sparse}}$ of the sparse kernel matrix and its regularization parameter ${\sigma }_{\text{jitter}}$ added to its diagonal elements can be set by the user.

GAP–SOAP implementation allows the inclusion of energy gradient information to the kernel matrix. In this case, the transformation matrix **L** has additional elements with the differentiation operators $\partial /\partial {M}_{\mathrm{at}}$, which results in calculating covariance between energies and their partial derivatives and also between derivatives [7, 26, 56].

The GAP software suite from the libatoms.org website contains a gap_fit program that trains the GAP model with the SOAP descriptor. The QUIP program is used to get predictions from the model trained by gap_fit. To use this combination for PES training and prediction, the data need to be formatted to extended XYZ format.

The gap_fit program provides tons of options that enable users to make fine adjustments to the training process, including settings for atomic energies’ offsets, sparsification, etc. (Table 2). However, the regularization hyperparameter and hyperparameters in the SOAP descriptor need to be set by the user manually, which makes it harder to realize the model’s full potential.

**Table 2:** Main tunable hyperparameters in the Gaussian approximation potential (GAP) model type and their corresponding keywords in the gap_fit program

Hyperparameter	Keyword	Description	Default values in MLatom^a^
$\upsigma$	default_sigma	List of regularization parameters for energy, force, viral and hessian	{0.0005, 0.001, 0.1, 0.1}
$\zeta$	zeta	Power of kernel	4
$\delta$	delta	Scaling of kernel	1
${r}_{\text{cut}}$	cutoff	Cutoff radius	6
${r}_{\Delta }$	cutoff_transition_width	Cutoff transition width	0.5
${n}_{\text{max}}$	n_max	Number of radial basis functions	6
${l}_{\text{max}}$	l_max	Number of angular basis functions	6
${\upsigma }_{\mathrm{atom}}$	atom_sigma	Gaussian smearing width of atom density	0.5

^a^Values chosen to provide reasonable accuracy for a small molecule (ethanol) by manual testing on the MD17 data set [48]

### TorchANI

TorchANI [28] is a Python library that implements the ANI model type [21], with PyTorch [58] as its backend for NN construction.

ANI is the abbreviation for ANAKIN-ME, which was back-engineered to Accurate NeurAl networK engINe for Molecular Energies. The ANI atomic environmental vector used in this model is a local descriptor, and is derived from the descriptor in Behler and Parrinello’s work [59].

The ANI descriptor ${\overrightarrow{G}}\!_{i}^{X}$ for element $X$’s $i$th atom contains a radial and an angular part, and both parts are further subdivided into subAEVs, in which the atoms taken into consideration will be limited by the same element.

For each element, the radial subAEV consists of input vector elements ${G}_{k}^{R}\in \mathbf{x}$ for different values of radial shift hyperparameters ${R}_{s}^{\left(k\right)}$:

60 $${G}_{k}^{R}=\sum_{j\ne i}{e}^{-\eta {\left({R}_{ij}-{R}_{s}^{\left(k\right)}\right)}^{2}}{f}_{c}\left({R}_{ij}\right).$$

Similarly, for each pair of elements, the angular subAEV consists of input vector elements ${G}_{p,q}^{A}\in \mathbf{x}$ for different combinations of angular shift hyperparameter ${\theta }_{s}^{\left(q\right)}$ and another set of radial shift hyperparameters ${R}_{s}^{\left(p\right)}$:

61 $${G}_{p,q}^{A}={2}^{1-\zeta }\sum_{j,k\ne i}{\left(1+\mathrm{cos}\left({\theta }_{ijk}-{\theta }_{s}^{\left(q\right)}\right)\right)}^{\zeta }{e}^{-\eta {\left(\frac{{R}_{ij}+{R}_{ik}}{2}-{R}_{s}^{\left(p\right)}\right)}^{2}}{f}_{C}\left({R}_{ij}\right){f}_{C}\left({R}_{ik}\right).$$

In the equations above, ${f}_{c}$ is the cutoff function used in Behler–Parrinello NN potentials [59] and similar to that used in GAP-SOAP, $\eta$, ${R}_{s}^{\left(k\right)}$,${R}_{s}^{\left(p\right)}$, ${\theta }_{s}^{\left(q\right)}$ and $\zeta$ are predefined hyperparameters. Parameters $\eta$ are defined separately for radial part and angular part similarly to ${R}_{s}$.

After being computed, each atom’s AEV will be plugged into its own NN as the input vector to predict atomic energy, and the atoms of the same element share the same NN structure to ensure the permutational invariance of the trained model.

The total energies are obtained by summing all atomic energies, while atomic forces are generated by differentiating atomic energies using PyTorch’s automatic differentiation engine. TorchANI reads HDF5 files, where training data are organized and stored.

As shown in Table 3, many hyperparameters can be tuned in ANI descriptors, not to mention the hyperparameters of NNs. However, as a Python library, TorchANI provides neither default values nor optimization method for hyperparameters, but only the basic core functions of ANI model type as building blocks. The final training scripts need to be written by the users themselves.

**Table 3:** Table of the main tunable hyperparameters in ANI model type related to the local AEV descriptor and their corresponding keywords in the TorchANI program

Hyperparameter	Keyword	Description	Default values in MLatom^a^
${R}_{\mathrm{C}}$ (radial)	Rcr	radial cutoff radius	5.3
${R}_{\mathrm{C}}$ (angular)	Rca	angular cutoff radius	3.5
$\eta$ (radial)	EtaR	radial smoothness in radial part	{16}
${R}_{\mathrm{s}}$ (radial)	ShfR	list of radial shifts in radial part	{0.90, 1.17, 1.44, 1.71, 1.98, 2.24, 2.51, 2.78, 3.05, 3.32, 3.59, 3.86, 4.12, 4.39, 4.66, 4.93}
$\eta$ (angular)	EtaA	radial smoothness in angular part	{8}
${R}_{\mathrm{s}}$ (angular)	ShfA	list of radial shifts in angular part	{0.90, 1.55, 2.20, 2.85}
${\theta }_{\mathrm{s}}$	ShfZ	list of angular shifts	{0.19, 0.59, 0.98, 1.37, 1.77, 2.16, 2.55, 2.95}
$\zeta$	Zeta	angular smoothness	{32}

Hyperparameters for neural networks are not listed

^a^Taken from the example script on the website of the program (https://aiqm.github.io/torchani-test-docs/examples/nnp_training.html)

### DeePMD-kit

DeePMD-kit [29] is a software with the Deep Potential Molecular Dynamics (DPMD) [30] ML model type and its successor Deep Potential—Smooth Edition (DeepPot-SE) [31] built-in. Like ANI, DPMD and DeepPot-SE are also based on NNs with local descriptors. Nevertheless, DeepPot-SE switched to a learned local descriptor rather than the fixed one in its predecessor.

In DeepPot-SE, the generalized local environment matrix $\widetilde{\mathbf{R}}^{i}$ (which is the descriptor of original DPMD) and the local embedding matrix ${\mathbf{G}}^{i}$ are used in representing the local environment of atom $i$ with ${N}_{i}$ neighboring atoms. The matrix $\widetilde{\mathbf{R}}^{i}$ has ${N}_{i}$ rows and each row are defined from relative coordinates and distances as:

62 $$\left\{s\left({r}_{ij}\right),\frac{{x}_{ij}}{{r}_{ij}}s\left({r}_{ij}\right),\frac{{y}_{ij}}{{r}_{ij}}s\left({r}_{ij}\right),\frac{{z}_{ij}}{{r}_{ij}}s\left({r}_{ij}\right)\right\},$$

where:

63 $$s\left({r}_{ij}\right)=\left\{\begin{array}{cc}\frac{1}{{r}_{ij}},& {r}_{ij}<{r}_{cs}\\ \frac{1}{{r}_{ij}}\left(\frac{1}{2}\mathrm{cos}\left(\pi \frac{{r}_{ij}-{r}_{cs}}{{r}_{c}-{r}_{cs}}\right)+\frac{1}{2}\right),& {r}_{cs}<{r}_{ij}<{r}_{c}\\ 0,& {r}_{ij}>{r}_{c}\end{array}\right.$$

The ${N}_{i}\times M$ matrix ${\mathbf{G}}^{i}$ is generated from the local embedding network (also called filter network), which outputs a $M$-dimensional vector for each neighboring atom $j$:

64 $${\mathbf{G}}_{jk}^{i}={g}_{k}\left(s\left({r}_{ij}\right)\right)$$

where ${g}_{k}$ is the $k$th output of local embedding network applied to $s\left({r}_{ij}\right)$.

The final descriptor, or the feature matrix ${\mathbf{D}}^{i}$ of atom $i$ is defined by

65 $${\mathbf{D}}^{i}={\left({\mathbf{G}}^{i1}\right)}^{*}{\widetilde{\mathbf{R}}}\!^{i}{\left({\widetilde{\mathbf{R}}}\!^{i}\right)}^{*}{\mathbf{G}}^{i2},$$

where ${\mathbf{G}}^{i1}$ and ${\mathbf{G}}^{i2}$ are first ${M}_{1}$ and ${M}_{2}$ columns of ${\mathbf{G}}^{i}$. The translational, rotational and permutational invariance is preserved in such expressions.

The feature matrices are then passed to NNs that generate atomic energies as the ANI model does.

DeePMD-kit program comes with its Python 3 environment, including TensorFlow and LAMMPS interface for MD simulations. The training data need to be saved in plain text in a specified style and then be transformed to what the program can read by the scripts they provide.

Training with the DeePMD-kit needs to be initialized with json input script, in which options and parameters are defined. The main tunable hyperparameters of DeepPot-SE are listed in Table 4, while hyperparameters in NNs (e.g., hyperparameters for network architecture, learning rate schedule, etc.) are not listed. However, this package cannot optimize those hyperparameters. Also, DeePMD-kit does not include in its native implementation the regularization scheme called *early stopping* often required in NN models to control the number of iterations performed during training, to stop the simulation before the model can reach an overfitting stage. Thus, we provide an external early stopping function as part of the interface module that monitors the training progress (based on the loss for the validation set) in the MLatom/DeePMD-kit output to stop the simulation when the criterion defined in the input has been reached.

**Table 4:** Main hyperparameters in DeepPot-SE model type, and their corresponding keywords in the DeePMD-kit program. Hyperparameters for neural networks are not listed

Hyperparameter	Keyword	Description	Default values in MLatom^a^
filter_neuron	filter_neuron	List of numbers of neurons in filter network	{30, 60}
${M}_{2}$	n_axis_neuron	Number of columns in ${\mathbf{G}}^{i2}$	6
n_neuron	n_neuron	List of numbers of neurons in fitting net	{80, 80, 80}
${r}_{c}$	rcut	Cutoff radius	6.5
${r}_{cs}$	rcut_smth	Radius cutoff transition starts	6.3
sel_a	sel_a	Maximum numbers of neighboring atoms	10 for each element

^a^Taken from [31]

### PhysNet

PhysNet [22] is another ML model type based on learned local descriptor but using a message-passing NN architecture as the underlying model.

In PhysNet, the embedding vectors ${\mathbf{e}}_{z}$ are used as the input vectors:

66 $${\mathbf{x}}_{i}^{0}={\mathbf{e}}_{{z}_{i}},$$

where the superscript $l$ over a vector denotes the layer number ($l=0$ stands for the input vector), and ${z}_{i}$ is the nuclear charge of atom $i$. Moreover, the number of features is defined by hyperparameter $F$.

Coordinates are transformed to $\mathbf{g}$ by applying $K$ radial basis functions and cutoff functions to internuclear distances:

67 $${g}_{k}\left({r}_{ij}\right)={f}_{c}\left({r}_{ij}\right)\cdot {e}^{-{\beta }_{k}{\left({e}^{-{r}_{ij}}-{\mu }_{k}\right)}^{2}},$$

where $\beta$ and ${\mu }_{k}$ are parameters of radial basis functions and *r*_ij_ denotes pairwise Euclidean distance between atoms *i* and *j*.

Then ${\mathbf{x}}^{0}$ is passed through a stack of ${N}_{\mathrm{module}}$ modules which are connected in series, and $\mathbf{g}$ is passed to each module.

There is a building block that will be repeatedly used in modules called a residual block. The residual block is a 2-layer mini residual neural network (ResNet), in which input vectors will also directly contribute to output vectors by skipping over the layers in between:

68 $${\mathbf{x}}_{i}^{l+2}={\mathbf{x}}_{i}^{l}+{\mathbf{W}}^{l+1}\sigma \left({\mathbf{W}}^{l}\sigma \left({\mathbf{x}}_{i}^{l}\right)+{\mathbf{b}}^{l}\right)+{\mathbf{b}}^{l+1},$$

where $\mathbf{W}$ and $\mathbf{b}$ consist of learnable weights and biases, $\sigma$ is the activation function.

Inside each module, a prototype vector $\widetilde{\mathbf{v}}$ will be generated by a NN first:

69 $${\widetilde{\mathbf{v}}}_{i}^{l}=\sigma \left({\mathbf{W}}_{\mathbf{I}}^{l}\sigma \left({\mathbf{x}}_{i}^{l}\right)+{\mathbf{b}}_{\mathbf{I}}^{l}\right)+\sum_{j\ne i}{\mathbf{G}}^{l}\mathbf{g}\left({r}_{ij}\right)\circ \sigma \left({\mathbf{W}}_{\mathbf{J}}^{l}\sigma \left({\mathbf{x}}_{j}^{l}\right)+{\mathbf{b}}_{\mathbf{J}}^{^{\prime}}\right),$$

where the elements of matrix $\mathbf{G}$ are learnable coefficients for ${g}_{k}\left({r}_{ij}\right)$, and $\circ$ is the Hadamard product operator.

This prototype $\widetilde{\mathbf{v}}$ then will be tuned by ${N}_{\mathrm{residual}}^{\mathrm{interaction}}$ residual blocks to get the optimized vector $\mathbf{v}$ which will then ‘interact’ with $\mathbf{x}$:

70 $${\mathbf{x}}_{i}^{l+1}={\mathbf{u}}^{l}\circ {\mathbf{x}}_{i}^{l}+{\mathbf{W}}^{l}\sigma \left({\mathbf{v}}_{i}^{l}\right)+{\mathbf{b}}^{l},$$

where $\mathbf{u}$ is also a learnable parameter.

After going through another ${N}_{\mathrm{residual}}^{\mathrm{atomic}}$ residual blocks, input $\mathbf{x}$ will be passed separately to the next module (if it exists) and the output block which turns $\mathbf{x}$ to module’s output ${\mathbf{y}}^{m}$, which contributes to the final output $\mathbf{y}$ after a linear transformation whose parameters are also learnable.

Unlike previously described models trained with local descriptors (GAP–SOAP, ANI, DeepPot-SE), PhysNet may also take long-range interactions (e.g., electrostatic and dispersion) into account. By default, dispersion corrections are enabled in the MLatom interface, while electrostatic corrections are disabled because their calculations require additional input (reference dipole moments).

The official implementation of the PhysNet model is programmed in Python 3.6 with TensorFlow v1, and the data need to be stored in the Numpy’s npz format of a specific structure. Similar to TorchANI, using the PhysNet program also needs much manual work on script-writing and hyperparameter-tuning (see Table 5 for the list of the main hyperparameters).

**Table 5:** Main tunable hyperparameters in PhysNet, and their corresponding keywords

Hyperparameter	Keyword	Description	Default values in MLatom^a^
$F$	num_features	Number of input features	128
$K$	num_basis	Number of radial basis functions	64
${N}_{\mathrm{module}}$	num_blocks	Number of modules	5
${N}_{\mathrm{ residual}}^{\mathrm{atomic}}$	num_residual_atomic	Number of residual blocks after interaction	2
${N}_{\mathrm{ residual}}^{\mathrm{interaction}}$	num_residual_interaction	Number of residual blocks in interaction	3
${N}_{\mathrm{ residual}}^{\mathrm{output}}$	num_residual_output	Number of residual blocks in output block	1
${r}_{\text{cut}}$	cutoff	Cutoff radius	10

^a^Taken from [22]

## Applications

In this section, we present several case studies demonstrating the capabilities of MLatom 2.

### Case Study 1: Hyperparameter Optimization

**Fig. 7:**
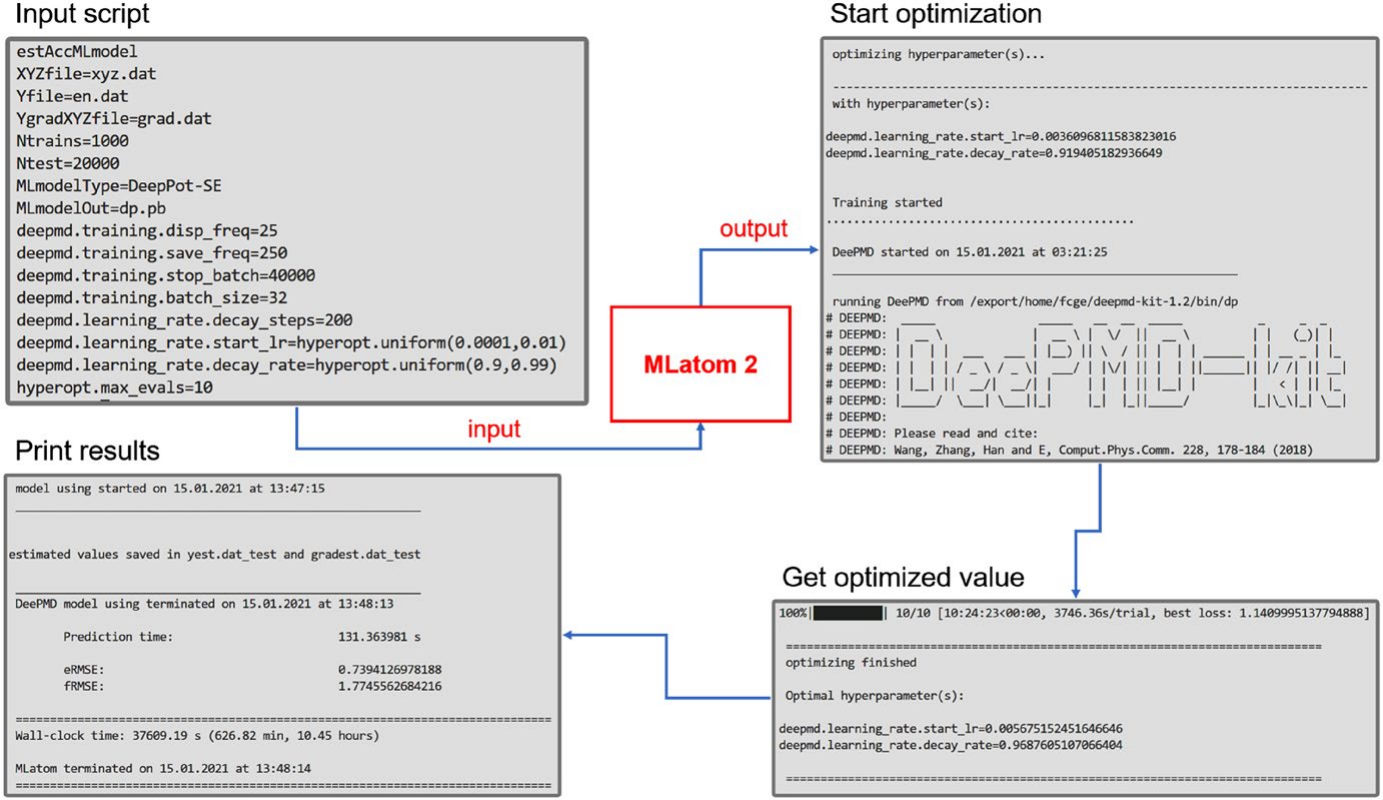
Part of input and output of MLatom for hyperparameter optimization of DeepPot-SE model using the interfaces to the hyperopt and DeePMD-kit packages

As mentioned in section "Hyperopt" in "Interfaces", a solution for hyperparameter optimization problem is given in MLatom 2 by introducing the interface to the hyperopt package. Here, we demonstrate a case using hyperopt interface to optimize the hyperparameters of the learning rate schedule in DeepPot-SE model type (start_lr and decay_rate, Table 6). For this, we used PES data (including energy gradients) of ethanol from MD17 data set [48]. A total of 1 k training points and 20 k test points were chosen randomly from the data set without overlapping. Other technical details can be found in Fig. 7 and Table 6.

**Table 6:** Root-mean-squares errors (RMSEs) in energies and energy gradients for DeepPot-SE potential of ethanol potential energy surface trained on 1 k random training points for the independent test set of 20 k randomly chosen test points for hyperparameters start_lr and decay_rate taken from the literature (Sets A^a^ and B^b^) and optimized using MLatom’s interfaces.^c^

	Set A from [31]^a^	Set B from [30]^b^	Optimized
start_lr (starting learning rate)	0.005	0.001	0.005675
decay_rate (decay rate)	0.96	0.95	0.9688
RMSE in energies (kcal/mol)	0.96	3.20	0.74
RMSE in gradients (kcal/mol/Å)	2.53	6.36	1.77

^a^Hyperparameters are taken for the DeepPot-SE model used for MD17 data set

^b^Hyperparameters are taken for the DPMD model used for MD17 data set

^c^In DeePMD-kit, a step decay schedule is used for learning rate decay. The related hyperparameter starting learning rate (start_lr) and the decay rate (decay_rate) were optimized, while the decay steps (decay_steps) were fixed to 200 with a stopping batch (stop_batch) set to 40,000. The search space was set to be from 0.0001 to 0.01 for starting learning rate and from 0.9 to 0.99 for the decay rate. Both spaces were set to be linear for 10 attempts of searching. The geometric mean of RMSE in energies and its gradients was used as the validation error

For comparison, we also took two sets of start_lr and decay_rate from original literature on the DeepPot-SE [31] and DPMD models [30] (see Table 6). These values were used for the same data set but with much larger training set sizes (50 and 95 k).

As shown in Table 6, the optimization process achieved significantly better accuracies in both energies and gradients prediction, despite being used for a related task as reported in the literature. This indicates that hyperparameter optimization is highly recommended for each new case, even for cases similar to previously published ones.

### Case Study 2: Learning Curves

**Fig. 8:**
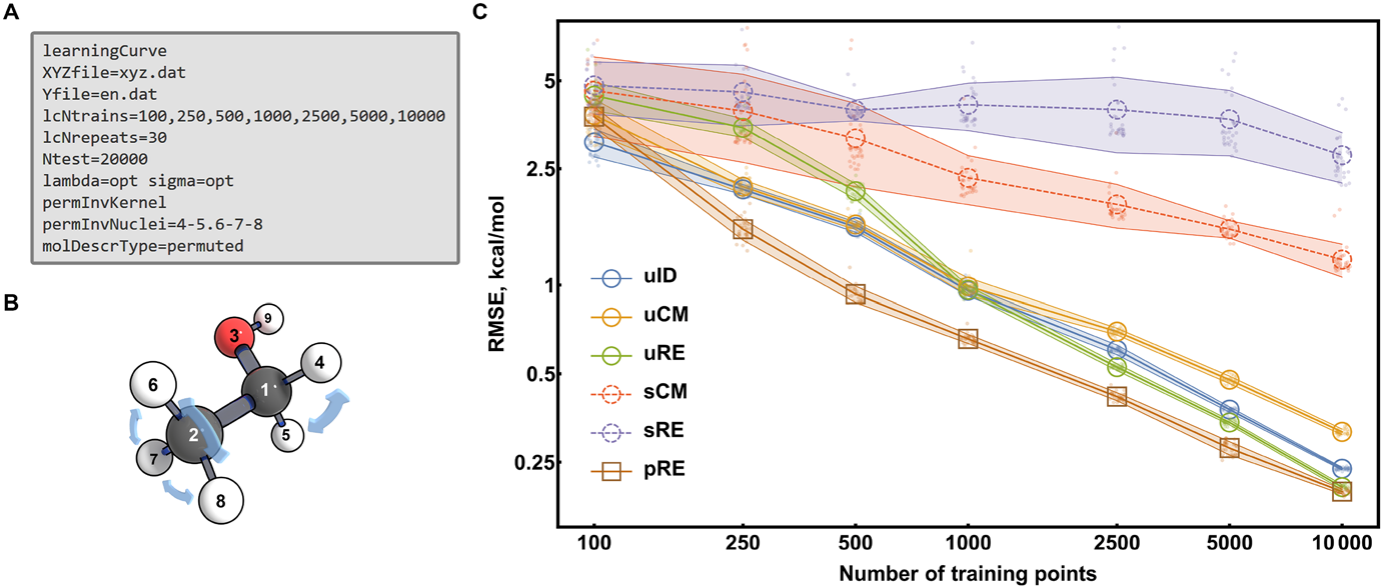
**a** Input file for learning curve task using the permuted RE descriptor with kernel ridge regression (KRR) (used with the Gaussian kernel, i.e., the ML model type is a permutationally invariant KREG). The scheme for the learning curve is defined with keywords *lcNtrains* and *lcNrepeats*. **b** A three-dimensional (3D) representation of an ethanol molecule. Atoms are numbered by their order in the MD17 data set [41]. Hydrogen atoms in methyl and methylene groups are permuted separately, as defined in the input using the option *permInvNuclei* = *4–5.6–7–8*. **c** Model performances with different descriptors and training set sizes. Hyperparameter optimization was performed throughout.* Markers* and* error bars* show the mean and standard deviation values of RMSEs in predictions for 20 k independent test points. All data sets were randomly sampled

In this part, we provide cases of MLatom’s learning curve task (see "Learning curves") to show how KRR performance varies with the different molecular descriptors being used. Unsorted, sorted, and permuted RE descriptors, unsorted and sorted Coulomb matrix, and unsorted inverse distances descriptor were examined on ethanol, with energy data from MD17 data set [48]. The descriptors are denoted as uRE, sRE, pRE, uCM, sCM, and uID, respectively, and details for RE and CM can be found in subsections "KREG" and "Coulomb matrix" in section "Native implementations". The uID descriptor is not among native implementations. Thus, it is provided to MLatom, which also demonstrates support for external, user-defined descriptors. We used the Gaussian kernel throughout, i.e., KRR with the uRE, sRE, and pRE descriptors are equivalent to the corresponding KREG model variants. All these descriptors were tested with seven training set sizes roughly evenly spaced on the log scale from 100 to 10 k. Other training and testing details can be found in Fig. 8a.

The results (Fig. 8b) show the big impact of molecular descriptor choice on ML performance. First of all, let us look at the unsorted descriptors. The RE descriptor and CM are both related to the unnormalized inverse distances (ID) (see subsections "Native implementations" and "sGDML"). The uRE descriptor is a normalized version of the uID descriptor, with its advantages and disadvantages manifested in differences in the corresponding learning curves. Normalization gives equal importance to both close-range and long-range interactions, which is detrimental to accuracy for scarce training data (up to 1 k training points in case of this data set) but is advantageous when more training data are available. uCM has a product of nuclear charges in the nominator, which is different for each pair of elements and may introduce sub-optimal weighting of the descriptor elements, leading to increased error relative to the uID descriptor [2].

The importance of properly taking into account the permutational invariance is demonstrated by using the sorted and permuted descriptors. Sorting is the simplest approach, but it causes discontinuities in the interpolant and leads to much worse results even compared to the unsorted descriptors. One of the more solid approaches, the permutationally invariant kernel using the permuted RE descriptors, can preserve permutational invariance without malfunctioning and achieves much better performance than uRE and better than uID (except for a very small number of training points).

### Case Study 3: Δ-Learning and Structure-Based Sampling

Training with Δ-learning [9] and choosing training points using structure-based sampling [5] can offer ML models with better accuracy. To showcase the superiority of these approaches, we compared them to the direct ML models of the target property and random sampling. Here, we provide test results with four combinations of learning (direct vs. Δ) and sampling (random vs. structure-based) approaches using the KREG model type. The data set from reference [9] containing the PES of CH_3_Cl calculated at several QM levels was used.

For the Δ-learning in this case study, CCSD(T)/CBS energy ${E}_{\mathrm{CCSD}(\mathrm{T})}$ served as the target energy, while MP2/aug-cc-pVQZ energy ${E}_{\mathrm{MP}2}$ was considered as the baseline energy. Thus, the Δ-learning model ${\Delta }_{\mathrm{MP}2}^{\mathrm{CCSD}(\mathrm{T})}$ is defined by:

71 $${\Delta }_{\mathrm{MP}2}^{\mathrm{CCSD}(\mathrm{T})}\left(\mathbf{x}\right)={E}_{\mathrm{MP}2}\left(\mathbf{x}\right)+\widehat{f}\left(\mathbf{x}\right)$$

where the ML model giving predictions $\widehat{f}\left(\mathbf{x}\right)$ is trained on the differences between target and baseline methods ${E}_{\mathrm{CCSD}(\mathrm{T})}-{E}_{\mathrm{MP}2}$. Thus, the cost of the Δ-learning model is determined by the cost of the baseline QC method, and the user should provide MLatom with the values calculated using the baseline QC method for both training and prediction.

**Fig. 9:**
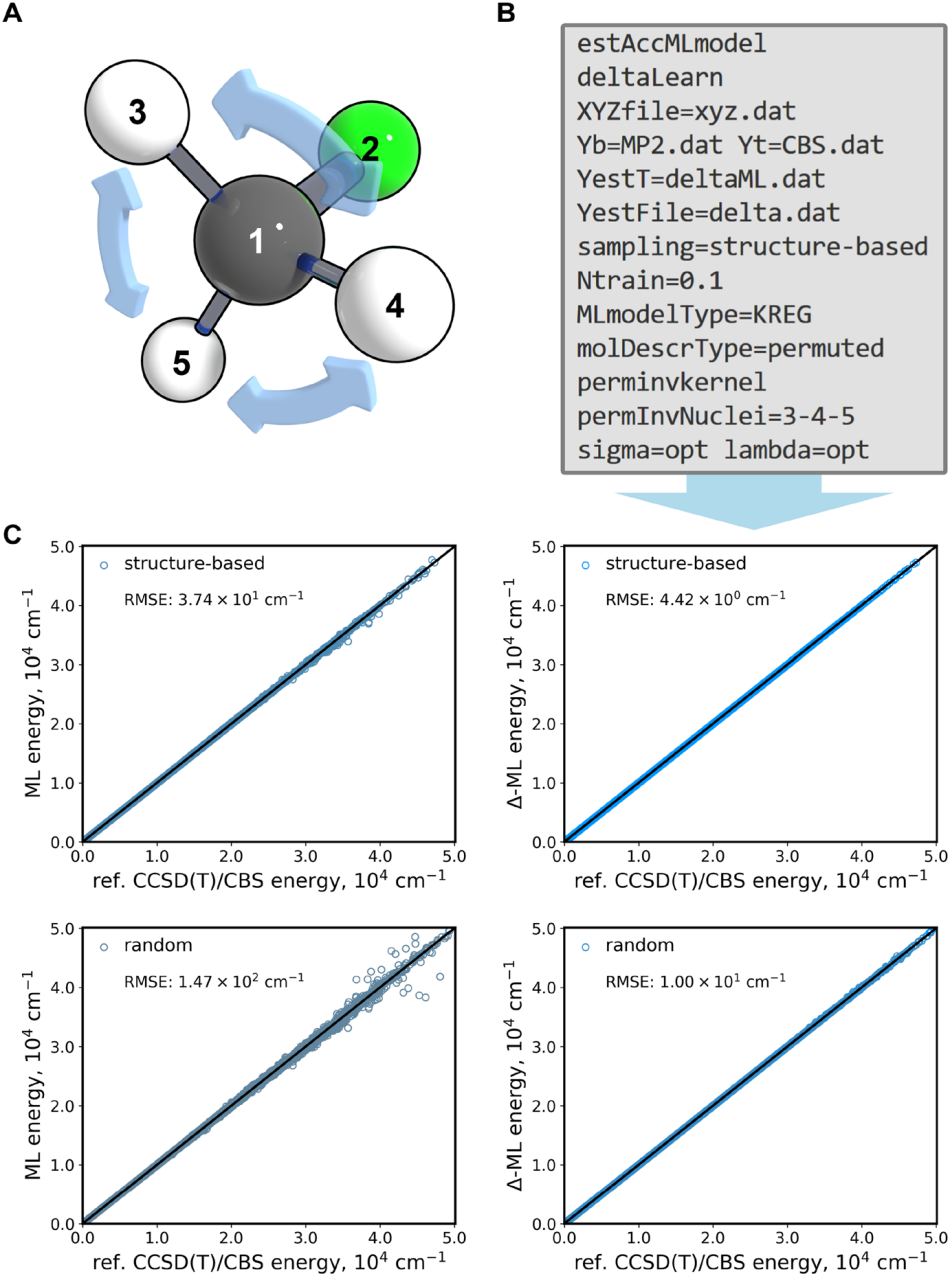
**a** A 3D representation of the CH_3_Cl molecule. Atoms are numbered by their order in the data set from reference [9]. Inside CH_3_Cl, hydrogen atoms 3, 4, and 5 are indistinguishable, and thus their permutations should result in no difference in molecular properties. **b** Sample input script for training ML models using the Δ-learning and structure-based sampling [5] for the selection of the training set. **c** ML energies vs. reference CCSD(T)/CBS energies. ML models were trained with the 10% points of the whole data set and were tested with the remaining 90% points. *R*^2^ is approaching 1 in all cases, with slightly larger values for more accurate models and thus are not shown for clarity.* Right column* Δ-learning models with MP2/aug-cc-pVQZ energies as a baseline.* Left column* ML model trained with reference CCSD(T)/CBS energies directly.* Bottom row* Data sets were split by random sampling.* Top row* Data sets were split by structure-based sampling

For sampling, we used 10% points of the whole data set as the training set and the remaining 90% as the test set. Also, as illustrated in Fig. 9a, the CH_3_Cl molecule has three indistinguishable hydrogen atoms, so the permutational invariant kernel was used (see "Permutationally invariant kernel").

Figure 9c shows a sample input file, and the scatter plots with RMSEs for all four combinations. Both Δ-learning and structure-based sampling led to much more accurate predictions for CCSD(T)/CBS energy than the simplest combination of random sampling and direct learning, and even more so when these two approaches were combined.

### Case Study 4: Absorption Spectrum

**Fig. 10:**
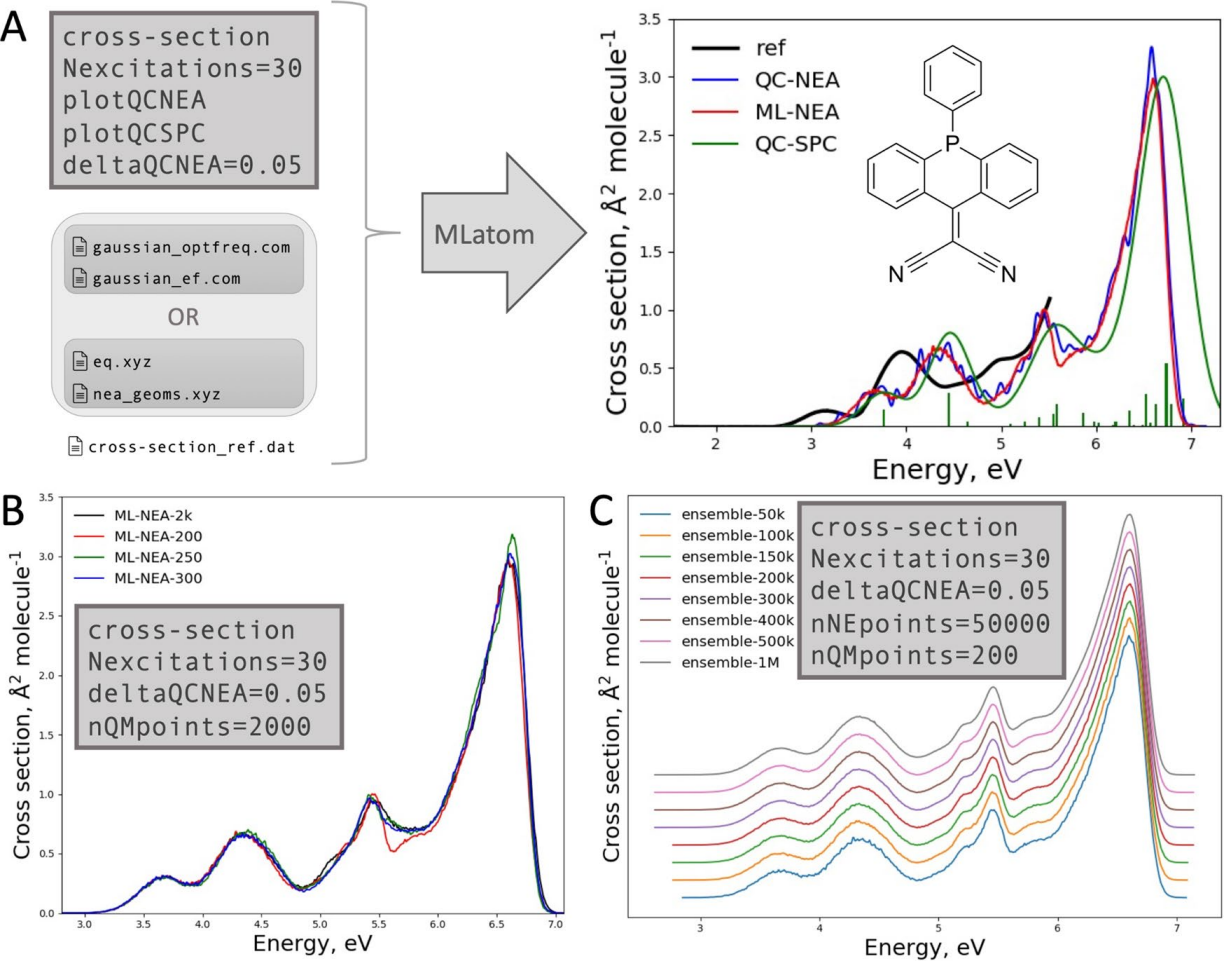
**a** Structure of the acridophosphine derivative molecule investigated here. MLatom input file and the list of additional required files for the ML-NEA calculations and the resulting spectrum. QC calculation details are defined in the Gaussian input files. Alternatively, the user can provide pre-calculated results (useful to refine spectra). The number of training points (200) was determined automatically by MLatom, and the resulting cross section ML-NEA is compared to the cross section obtained using traditional QC-NEA with the same points and single-point convolution approach (QC-SPC) based on broadening lines only for the equilibrium geometry. The broadening factor for QC-NEA is 0.05 eV and for QC-SPC 0.3 eV. The reference (ref) spectrum is the experimental cross section from [60]. **b** ML-NEA spectra with sample input file for 200, 250, 300, and 2 k training points. **c** Sample input file and spectra calculated with 50 k, 100 k, 150 k, 200 k, 300 k, 400 k, 500 k, and 1 M points in the nuclear ensemble. The spectra are shifted vertically for clarity

In this case study, we will calculate the absorption cross section for an acridophosphine derivative [60] (Fig. 10) using ML-NEA implementation in MLatom, and discuss the effect of the number of points in the training set and nuclear ensemble. MLatom allows refining cross sections using existing data. Therefore, we used this feature to perform all the simulations using QC data at the ωB97XD[61]/def2-SVP [62–64] level of theory from our previous publication (see [60] for computational details; energies and oscillator strengths for 30 excited states are available at https://doi.org/10.6084/m9.figshare.13635353).

By default, MLatom determines the optimal training point number iteratively by adding 50 points at each step, and the cross section is calculated using 50 k in the nuclear ensemble. In our example, the ML-NEA procedure converged after 200 points. For comparison, the cross section obtained without ML using these 200 points in the nuclear ensemble (QC-NEA spectrum, Fig. 10a) curve has many peaks, and it is hard to judge what are the actual peaks and what are the artifacts of insufficient statistical sampling, while the ML-NEA spectrum is much smoother and ML calculations incur only small additional computational cost. The popular single-point convolution (SPC) approach gives a blue-shifted spectrum with an arbitrary bandwidth [65].

Although MLatom determines the training set size automatically, one can always request calculations of additional training points to check whether the spectrum has “truly converged” by visual inspection. For example, by comparing ML-NEA spectra with the ones calculated using 200, 250, and 300 points, one can see that they are very close to each other with some minor deviations (Fig. 10b). The ML-NEA spectrum obtained with 300 points is nearly the same as the spectrum calculated with 2 k points. One should remember, however, that the accuracy of ML-NEA depends on the accuracy of the underlying QC method, and the difference between the experimental spectrum and ML-NEA, even with the largest 2 k number of training points, is bigger than the difference between ML-NEA spectra with 200, 250, and 300 points (compare Fig. 10a, b). One minor aspect is that, although the default 50 k points is a rather large ensemble, it still leads to slightly rugged curves. Perfectly smooth curves can be obtained by simply increasing the number of ensemble points (Fig. 10c), and MLatom is a computationally efficient tool for this task; one can calculate very smooth curves, e.g., with 1 M points. One should keep in mind though, that such a large number is not necessary, and it will take about 10 GB of disk space to store the file with the nuclear ensemble geometries and take more time to make ML prediction and cross section calculations.

## Conclusions

In this review article, we have described the MLatom 2 software package, which provides an integrative platform for AML simulations. Unlike other specialized AML packages, MLatom has been developed with the aim of facilitating the application of ML models to the wide variety of tasks often required in computational chemistry research.

Its capabilities range from native implementations such as the KREG model and other KRR model types (with the Coulomb matrix or any other user-defined descriptors as well as the Gaussian, Matérn, Laplacian, and exponential kernel functions) to interfaces to the third-party packages with popular models. The latter models are overviewed here for the sake of completeness and include sGDML, GAP–SOAP, ANI, DPMD, DeepPot-SE, and PhysNet. Other AML model types can be implemented easily by using the modular approach adopted in MLatom for third-party interfaces.

Other important features of MLatom for AML simulations such as model evaluation, hyperparameter optimization, sampling procedures (including farthest-point and structure-based sampling), Δ-learning, self-correction, and automatic learning curve generation are overviewed too. We also discussed how all steps required for the absorption spectrum simulation with the machine learning-nuclear ensemble approach (ML-NEA) are integrated in MLatom. Finally, we provided examples of how MLatom can be used for selected applications: hyperparameter optimization, learning curve generation, Δ-learning and structure-based sampling, and absorption spectrum simulation.

MLatom provides a user-friendly, integrated platform for researchers who want to use a wide variety of AML approaches and related techniques. It is also a useful package for educational purposes as it is used for teaching the basic and advanced concepts behind ML use in quantum chemistry (see, e.g., the book chapter [2], and online tutorial at http://MLatom.com). We are continually developing this platform based on the needs for practical AML computations such as dynamics, calculation of excited-state properties, and rovibrational spectrum simulations, improvement of QC methods, and materials design.

## Data Availability

All the data used in this manuscript are available from literature and online databases as cited in the article. No new data was generated in this study.

## Code Availability

MLatom 2 is available from http://MLatom.com free of charge for non-commercial and non-profit uses, such as academic research and education. All interfaced third-party software should be obtained and installed by the user separately.
